# Elastodynamic Behaviour of Laminate Structures with Soft Thin Interlayers: Theory and Experiment

**DOI:** 10.3390/ma15041307

**Published:** 2022-02-10

**Authors:** Maria V. Wilde, Mikhail V. Golub, Artem A. Eremin

**Affiliations:** 1 Faculty of Mathematics and Mechanics, Saratov State University, 410012 Saratov, Russia; mv_wilde@mail.ru; 2 Institute for Mathematics, Mechanics and Informatics, Kuban State University, 350040 Krasnodar, Russia; m_golub@inbox.ru

**Keywords:** laminate, soft material, thin interlayer, guided waves, edge waves, effective boundary conditions

## Abstract

Laminate structures composed of stiff plates and thin soft interlayers are widely used in aerospace, automotive and civil engineering encouraging the development of reliable non-destructive strategies for their condition assessment. In the paper, elastodynamic behaviour of such laminate structures is investigated with emphasis on its application in ultrasonic based NDT and SHM for the identification of interlayer mechanical and interfacial contact properties. A particular attention is given to the practically important frequency range, in which the wavelength considerably exceeds the thickness of the film. Three layer model with spring-type boundary conditions employed for imperfect contact simulation is used for numerical investigation. Novel effective boundary conditions are derived via asymptotic expansion technique and used for analysis of the peculiar properties of elastic guided waves in considered laminates. It is revealed that the thin and soft film influences the behaviour of the laminate mainly via the effective stiffnesses being a combination of the elastic moduli of the film, its thickness and interface stiffnesses. To evaluate each of these parameters separately (or to figure out that the available experimental data are insufficient), a step-wise procedure employing the effective boundary conditions is proposed and tested versus the laser Doppler vibrometry data for Lamb waves in Aluminium/Polymer film/Alumunium structure. A good agreement between theoretical and experimental data is demonstrated for a certain symmetric laminate specimen. The possibility of using film-related thickness resonance frequencies to estimate the film properties and contact quality is also demonstrated. Additionally, the rich family of edge waves is also investigated, and the splitting of fundamental edge waves into pairs is revealed.

## 1. Introduction

Laminate thin-walled structures composed of stiff plates and soft polymeric interlayers are typical for many industrial applications. Among such examples are adhesively bonded metallic or fibre-reinforced composite components widely used in aerospace and automobile manufacturing providing an adequate compromise between weight reduction, strength properties and cost-efficient assembling [1,2] or laminated glass consisting of a plastic interlayer surrounded by two adjacent glass plates which have become a ubiquitous solution for automobile windshields and in architectural glazing due to its impact-energy absorbing properties [3].

Since the integrity of the bonds in multi-layered assemblies directly affects the product quality, development and implementation of reliable non-destructive strategies for their condition assessment are essential [4,5]. Together with conventional ultrasonic testing [6], the approaches employing elastic guided waves (EGWs) as a physical basis are emerging to characterize adhesive joint properties [7,8,9,10]. For visualization of localized macroscopic damage in bonded structures with EGWs, non-contact imaging techniques are being developed based on object surface scanning with a laser source [11], laser interferometer [12] and their combinations [13]. Since guided wave dispersion properties strongly depend on structural material parameters, EGWs are valuable for global assessment of adhesive bonding integrity [8,14] and might be also used for the estimation of adhesive mechanical properties [15,16]. The latter is particularly important for in- and post-manufacturing quality control of bonded structures because the strength properties of adhesives strongly depend on curing conditions [17,18].

In the EGWs diagnostics of laminate assemblies with soft interlayers, relevant mathematical and computational models describing their elastodynamic behaviour are essential for adequate interpretation of the experimental data. A natural and basic way is to model such waveguides as multilayered objects assuming continuity conditions for displacement and stress components at the interlaminar interfaces [15] (for bonded structures, they are known as tri-layer models). To handle possible imperfectness in interface coupling, such models are further modified by including an additional viscoelastic interfacial layer [19] or assuming the spring-type boundary conditions (SBCs) between the laminae [8,20].

When the thickness of internal soft layers is sufficiently small compared to the wavelength, their dynamics might be neglected and replaced by certain effective boundary conditions (EBCs) coupling two external laminae and tuned to address interlayer mechanical properties and the contact quality. As EBCs, uniformly distributed SBCs working in traction/compression and shear are widely used [21,22] (i.e., if adhesively bonded structures are considered this relates to both cohesive and adhesive properties of bonded joints). Alternatively, more sophisticated and precise models are proposed based on asymptotic expansion techniques using a small parameter related to the thickness of the interlayer [23,24,25]. However, to our best knowledge, up to now these models did not take into account contact quality and their accuracy was not higher than the first order of the small parameter.

Compared to Lamb waves (LWs) in a monolithic single lamina, the presence of a soft polymeric insert and layering of the waveguide sufficiently complicates corresponding EGW phenomena. Theoretical considerations reveal that Lamb-type EGWs propagating in such laminates are composed of modes that could be associated with corresponding LWs of sublayers and coupling modes related to the global structure [19]. Moreover, certain of the former are disparted in the laminate structure into mode pairs, which dispersion curves in broad frequency ranges traverse along corresponding trajectories of these LWs [20,26]. Finally, specific mode repulsion phenomena, not occurring in a monolithic layer, could be also pronounced [27]. It is observed that all these peculiarities of EGWs could depend both on mechanical properties of thin interlayers and contact quality between laminae [8,19]. Thus, a thorough investigation of corresponding EGW phenomena and understanding of their dependencies from the aforementioned input data is essential for the reliable application of EGWs for the evaluation of laminate structures.

The aim of the current study is to comprehensively investigate and explain the influence of thin and soft interlayers on the behaviour of EGWs in laminate isotropic structures with particular emphasis on the application of the obtained results for the identification of mechanical properties of such sublayers and evaluation of interlaminar contact integrity. For this purpose, extensive numerical analysis of EGW characteristics in a three-layered geometrically symmetric laminate with a thin film is performed while elastic constants and thickness of the latter as well as contact quality are serving as input. Along with the well-established tri-layer model enriched with SBCs between the laminae to handle possible contact degradation [7,8,20], a novel asymptotic model for the considered layered structure is proposed and the corresponding EBCs are derived. Employing them, it becomes possible to explain rigorously the nature of mode pairs observed numerically by Loukkal et al. [19], Mezil et al. [20], Puthillath et al. [26] and to derive a clear semi-analytical representation for the thickness resonance frequencies of the laminate. Moreover, these EBCs allow advancing results of Gauthier et al. [8] on bonding quality assessment by estimating specific frequency ranges and particular EGWs, where and on which the influence of interlayer mechanical properties and its bonding quality with external lamina is the most pronounced. Using this information, a preliminary guideline for interlayer identification is proposed and tested over available experimental data making it of potential interest for ultrasonic based NDT/SHM. As a first step to further development of EGWs-based techniques, edge waves (EWs) in the laminate with thin soft interlayers are investigated and the complete picture of EGWs is presented. From the practical point of view, the EWs can be used to detect a weakening of the bond, localized near the edge. Moreover, together with LWs and SH-waves, they could provide additional information for identification of mechanical properties of the film and its contact conditions.

## 2. Mathematical Modelling

### 2.1. Exact Statement of Boundary Value Problem

**Figure 1:**
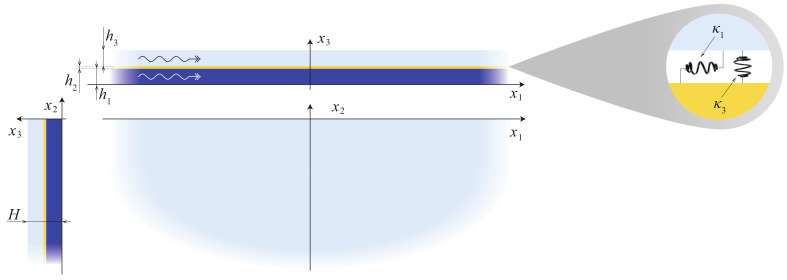
Geometry of the problem.

Let us consider a laminate composed of two isotropic and homogeneous elastic layers $D_{1}$ and $D_{3}$ of thicknesses $h_{1}$ and $h_{3}$ and a thin film $D_{2}$ of thickness $h_{2}$ between them as shown in Figure 1. Materials of the layers are characterized by the mass density $\rho_{q}$, Young’s modulus $E_{q}$ and Poisson ratio $\nu_{q}$ (q=1,2,3).

The stress tensor components $\sigma_{lm}^{\left(q\right)}$ (l,m=1,2,3) and the displacement vector $u^{\left(q\right)}=\left\{u_{1}^{\left(q\right)},u_{2}^{\left(q\right)},u_{3}^{\left(q\right)}\right\}$ in all the layers satisfy governing equations of linear elastodynamics

(1) $$\frac{\partial \sigma_{lm}^{\left(q\right)}}{\partial x_{m}}-\rho_{q}\frac{\partial^{2}u_{l}^{\left(q\right)}}{\partial t^{2}}=0,$$

where $x_{m}$ are Cartesian coordinates (see Figure 1), *t* is the time. Let us assume that the materials of all the layers are isotropic and obey the Hooke’s law. The stress tensor components can be expressed through the ones of the displacement vector as follows:

(2) $$\sigma_{lm}^{\left(q\right)}=\lambda_{q}\nabla \cdot u^{\left(q\right)}\delta_{lm}+\mu_{q}\left(\frac{\partial u_{l}^{\left(q\right)}}{\partial x_{m}}+\frac{\partial u_{m}^{\left(q\right)}}{\partial x_{l}}\right),$$

where $\delta_{lm}$ is Kronecker’s delta, $\lambda_{q}$, $\mu_{q}$ are Lamé constants. Let us introduce parameter

$$\beta_{q}^{2}=\frac{c_{q,T}^{2}}{c_{q,L}^{2}}=\frac{\mu_{q}}{\lambda_{q}+2\mu_{q}}=\frac{1-2\nu_{q}}{2(1-\nu_{q})},$$

where

$$c_{q,L}=\sqrt{\frac{\lambda_{q}+2\mu_{q}}{\rho_{q}}},$$

$$c_{q,T}=\sqrt{\frac{\mu_{q}}{\rho_{q}}}$$

are the velocities of the longitudinal and transverse waves, respectively.

The SBCs connecting the displacement vector $u^{\left(q\right)}$ and the traction vector composed of tangential and normal stresses $\tau^{\left(q\right)}=\left\{\sigma_{13}^{\left(q\right)},\sigma_{23}^{\left(q\right)},\sigma_{33}^{\left(q\right)}\right\}$ at the internal interfaces $x_{3}=z_{p}=\sum_{i=1}^{p}h_{i}$ (p=1,2) are written following [28,29,30,31]:

(3) $$\tau^{\left(p+1\right)}=\tau^{\left(p\right)}=\varkappa^{\left(p\right)}\left(u^{\left(p+1\right)}-u^{\left(p\right)}\right),\;x_{3}=z_{p}.$$

At the outer surfaces of the waveguide, stress-free boundary conditions (BCs)

(4) $$\tau^{\left(1\right)}=0,\;x_{3}=0;\;\;\tau^{\left(3\right)}=0,\;x_{3}=H$$

are assumed.

The components of the stiffness matrices $\varkappa^{\left(p\right)}$ have the form

$$\varkappa_{lm}^{\left(p\right)}=\varkappa_{l}^{\left(p\right)}\delta_{lm}.$$

The dispersion relation describing plane GWs propagating in an infinite multi-layered laminate can be obtained by reducing of the problem stated above to the plane one (LWs) or anti-plane one (SH-waves) in the plane, say, ($x_{1},x_{3}$). After the application of the Fourier transform with respect to $x_{1}$ coordinate and the Laplace transform with respect to the time *t* [32], governing Equation (1) are reduced to the system of ordinary differential equations for each layer with respect to $x_{3}$ and with the wavenumber *k* and the circular frequency ω=2πf as parameters. The solution of the system of differential equation is then substituted into the transformed BCs (3) and (4). As a result, an eigenvalue problem is formulated, which is reduced to the dispersion equation

D(k,ω)=0

and solved numerically following [33]. Table 1 presents the material properties used further for numerics.

### 2.2. Modeling of the Film via EBCs

Let us suppose that the EGWs, guided by the laminate described in Section 2.1, satisfy the condition $L\gg h_{2}$, where *L* is the characteristic wavelength. In this case, the problem stated in Section 2.1 can be reduced to a simplified one for a two-layered plate, composed of layers $D_{1}$ and $D_{3}$ with some effective boundary conditions (EBCs) on the interface between them, representing approximately the influence of the thin film. Using of asymptotic methods is a natural way to construct such conditions. In [23,24], the EBCs were obtained by asymptotic expansion of the transfer matrix of the interlayer. In [25], an another approach is used, involving expansion of displacements and stresses both in the interlayer and in the surrounding medium. A review of a various approaches to modeling thin layers via EBSc can also be found in [25]. Here we present an alternative method for deriving EBCs, which employs the asymptotic integration of Equations (1) and (2) for the film. This approach is based on the ideas of Kaplunov et al. [38], which were used in [39] to construct the EBCs for a half-space coated by a thin film.

Let us shift the origin of coordinate $x_{3}$ to the middle surface of the film by setting $y=x_{3}-\left(h_{1}+h_{0}\right)$ with $h_{0}=h_{2}/2$, then the internal interfaces $x_{3}=z_{p}$ correspond to $y=\pm h_{0}$. For the boundary values of traction components and displacements of the external layers, we introduce notations

$${\hat{\sigma }}_{l3}^{\left(\pm 1\right)}={\left.\sigma_{l3}^{\left(2\pm 1\right)}\right|}_{y=\pm h_{0}},\;\;{\hat{u}}_{l}^{\left(\pm 1\right)}={\left.u_{l}^{\left(2\pm 1\right)}\right|}_{y=\pm h_{0}}.$$

With the new notations, BCs (3) can be written in the form

(5) $${\left.\sigma_{l3}^{\left(2\right)}\right|}_{y=\pm h_{0}}={\hat{\sigma }}_{l3}^{\left(\pm 1\right)},\;{\left.u_{l}^{\left(2\right)}\right|}_{y=\pm h_{0}}={\hat{u}}_{l}^{\left(\pm 1\right)}\mp \xi_{l}^{\left(\pm 1\right)}{\hat{\sigma }}_{l3}^{\left(\pm 1\right)},$$

where $\xi_{l}^{\left(\pm 1\right)}={\left[\varkappa_{l}^{\left(\frac{3\pm 1}{2}\right)}\right]}^{-1}$ are interface compliances. Let μ and ρ be the characteristic values of the shear moduli and densities of the external layers. The behaviour of the film can be described in terms of dimensionless parameters $\varepsilon =h_{0}/L$, $\mu_{2}/\mu$, $\rho_{2}/\rho$. In order to consider the general case of long-wave vibrations, we assume ε≪1, $\mu_{2}/\mu ∼1$, $\rho_{2}/\rho ∼1$ and introduce dimensionless variables

(6) $$\eta_{i}=\frac{x_{i}}{L},\;\zeta =\frac{x_{3}}{h_{0}},\;\tau =\frac{tc_{T}}{L},\;u_{l}^{\left(2\right)}=\frac{h_{0}\mu }{\mu_{2}}w_{l},\;\sigma_{lm}^{\left(2\right)}=\mu p_{lm}.$$

Here and further on i,j=1,2,i≠j, $c_{T}=\sqrt{\mu /\rho }$. Let us write down BCs (5) in dimensionless variables (6) as the sum

(7) $${\left.p_{l3}\right|}_{\zeta =1}+{\left.p_{l3}\right|}_{\zeta =-1}=\frac{2}{\mu }S_{l3},\;{\left.w_{l}\right|}_{\zeta =1}+{\left.w_{l}\right|}_{\zeta =-1}=\frac{2\mu_{2}}{h_{0}\mu }{\tilde{U}}_{l}$$

and the difference

(8) $$\begin{array}{cc} \; & {\left.p_{l3}\right|}_{\zeta =1}-{\left.p_{l3}\right|}_{\zeta =-1}=\frac{1}{\mu }\left({\hat{\sigma }}_{l3}^{\left(1\right)}-{\hat{\sigma }}_{l3}^{\left(-1\right)}\right),\\ \; & {\left.w_{l}\right|}_{\zeta =1}-{\left.w_{l}\right|}_{\zeta =-1}=\frac{\mu_{2}}{h_{0}\mu }\left({\hat{u}}_{l}^{\left(1\right)}-{\hat{u}}_{l}^{\left(-1\right)}-P_{l},\right), \end{array}$$

where

(9) $$\begin{array}{c} S_{l3}=\frac{1}{2}\left({\hat{\sigma }}_{l3}^{\left(1\right)}+{\hat{\sigma }}_{l3}^{\left(-1\right)}\right),\;U_{l}=\frac{1}{2}\left({\hat{u}}_{l}^{\left(1\right)}+{\hat{u}}_{l}^{\left(-1\right)}\right),\;{\tilde{U}}_{l}=U_{l}-Q_{l},\\ Q_{l}=\frac{1}{2}\left(\xi_{l}^{\left(1\right)}{\hat{\sigma }}_{l3}^{\left(1\right)}-\xi_{l}^{\left(-1\right)}{\hat{\sigma }}_{l3}^{\left(-1\right)}\right),\;P_{l}=\xi_{l}^{\left(1\right)}{\hat{\sigma }}_{l3}^{\left(1\right)}+\xi_{l}^{\left(-1\right)}{\hat{\sigma }}_{l3}^{\left(-1\right)}. \end{array}$$

After substituting (6) into (1) and (2), one can rewrite this system in the form

(10) $$\begin{array}{cc} \; & \frac{\partial p_{33}}{\partial \zeta }=-\varepsilon \left(\partial_{i}p_{i3}+\partial_{j}p_{j3}\right)+\varepsilon^{2}\partial_{\tau }^{2}w_{3},\\ \; & \frac{\partial p_{i3}}{\partial \zeta }=-\varepsilon \left(1-2\beta_{2}^{2}\right)\partial_{i}p_{33}-\varepsilon^{2}\left[\partial_{\mathrm{pl}}^{2}w_{i}+\left(3-4\beta_{2}^{2}\right)\partial_{i}\partial_{j}w_{j}\right],\\ \; & \frac{\partial w_{3}}{\partial \zeta }=\beta_{2}^{2}p_{33}-\varepsilon \left(1-2\beta_{2}^{2}\right)\left(\partial_{i}w_{i}+\partial_{j}w_{j}\right),\;\frac{\partial w_{i}}{\partial \zeta }=p_{i3}-\varepsilon \partial_{i}w_{3} \end{array}$$

and

(11) $$\begin{array}{cc} \; & p_{ii}=\left(1-2\beta_{2}^{2}\right)p_{33}+2\varepsilon \left[2\left(1-\beta_{2}^{2}\right)\partial_{i}w_{i}+\left(1-2\beta_{2}^{2}\right)\partial_{j}w_{j}\right],\\ \; & p_{ij}=\varepsilon \left(\partial_{j}w_{i}+\partial_{i}w_{j}\right), \end{array}$$

where $\partial_{i}=\frac{\partial }{\partial \eta_{i}}$, $\partial_{\tau }^{2}=\frac{\rho_{2}\mu }{\rho \mu_{2}}\frac{\partial^{2}}{\partial \tau^{2}}$, $\partial_{\mathrm{pl}}^{2}=4\left(1-\beta_{2}^{2}\right)\partial_{i}^{2}+\partial_{j}^{2}-\partial_{\tau }^{2}$. Operator $\partial_{\mathrm{pl}}^{2}$ is related to the theory of plate extension (see below).

Let us assume that the functions $p_{l3}$, $w_{l}$ and their derivatives with respect to $\eta_{i}$ and τ have the order of unity as ε→0. Under this assumption, system (10) can be integrated by making use of asymptotic iterations [38]. In the zero order approximation, we omit in (10) all the terms with ε and $\varepsilon^{2}$. Then the system (10) gives

(12) $$\begin{array}{cc} \; & p_{33}^{0}=s_{33}+O\left(\varepsilon \right),\;p_{i3}^{0}=s_{i3}+O\left(\varepsilon \right),\\ \; & w_{3}^{0}=v_{3}+\beta_{2}^{2}s_{33}\zeta +O\left(\varepsilon \right),\;w_{i}^{0}=v_{i}+s_{i3}\zeta +O\left(\varepsilon \right), \end{array}$$

where $s_{l3}$, $v_{l}$ are arbitrary functions of $\eta_{i}$ and τ. By substituting (12) in the sum of BC (7), we find

(13) $$s_{l3}=\frac{1}{\mu }S_{l3},\;v_{l}=\frac{\mu_{2}}{h_{0}\mu }{\tilde{U}}_{l}.$$

Now we are in the position to obtain the first order approximation. By restoring the terms with ε and substituting into them already known zero order approximation, we come again to the system, which allows integration, and so on. It is convenient to write integrals of the odd functions of ζ as even functions, which turns to zero at ζ=±1 (e.g., the integral of ζ is $\left(\zeta^{2}-1\right)/2$). Then formulae (13) are valid for any approximation. After constructing approximation of $p_{l3}^{n}$, $v_{l}^{n}$ of desirable order *n*, one can substitute them in the difference of BCs (8) and obtain the relations between the boundary values ${\hat{\sigma }}_{l3}^{\left(\pm 1\right)}$, ${\hat{u}}_{l}^{\left(\pm 1\right)}$, i.e., the EBCs. Besides, we have the formulae for all the displacements and stresses in the film (for $p_{ii}$, $p_{ij}$, formulae (11) must be used). As soon as the problem for the domains $D_{1}$, $D_{3}$ connected via the EBCs is solved, one can reconstruct the distribution of the stresses and the displacement in the film with the asymptotic error $O\left(\varepsilon^{n+1}\right)$.

In the original variables, the EBCs of the second order have the form

(14) $$\begin{array}{cc} \; & {\hat{\sigma }}_{33}^{\left(1\right)}-{\hat{\sigma }}_{33}^{\left(-1\right)}=-j_{1}h_{2}\left(\frac{\partial S_{i3}}{\partial x_{i}}+\frac{\partial S_{j3}}{\partial x_{j}}\right)+j_{2}h_{2}\rho_{2}\frac{\partial^{2}{\tilde{U}}_{3}}{\partial t^{2}},\\ \; & {\hat{\sigma }}_{i3}^{\left(1\right)}-{\hat{\sigma }}_{i3}^{\left(-1\right)}=-j_{1}h_{2}\left(1-2\beta_{2}^{2}\right)\frac{\partial S_{33}}{\partial x_{i}}-j_{2}h_{2}\mu_{2}\left[\Omega_{\mathrm{pl}}{\tilde{U}}_{i}+\left(3-4\beta_{2}^{2}\right)\frac{\partial^{2}{\tilde{U}}_{j}}{\partial x_{i}\partial x_{j}}\right],\\ \; & {\hat{u}}_{3}^{\left(1\right)}-{\hat{u}}_{3}^{\left(-1\right)}=\frac{h_{2}}{\lambda_{2}+2\mu_{2}}S_{33}+P_{3}-j_{1}h_{2}\left(1-2\beta_{2}^{2}\right)\left(\frac{\partial {\tilde{U}}_{i}}{\partial x_{i}}+\frac{\partial {\tilde{U}}_{j}}{\partial x_{j}}\right)-j_{2}\frac{h_{2}^{3}\beta_{2}^{2}}{12\mu_{2}}\Omega_{3}S_{33},\\ \; & {\hat{u}}_{i}^{\left(1\right)}-{\hat{u}}_{i}^{\left(-1\right)}=\frac{h_{2}}{\mu_{2}}S_{i3}+P_{i}-j_{1}h_{2}\frac{\partial {\tilde{U}}_{3}}{\partial x_{i}}+j_{2}\frac{h_{2}^{3}}{12\mu_{2}}\left[\Omega_{i}S_{i3}+\left(1-\beta_{2}^{2}\right)\frac{\partial^{2}S_{j3}}{\partial x_{i}\partial x_{j}}\right], \end{array}$$

where

(15) $$\begin{array}{cc} \; & \Omega_{\mathrm{pl}}=\underline{4\left(1-\beta_{2}^{2}\right)\frac{\partial^{2}}{\partial x_{i}^{2}}+\frac{\partial^{2}}{\partial x_{j}^{2}}}-\frac{1}{c_{2,T}^{2}}\frac{\partial^{2}}{\partial t^{2}},\;\Omega_{3}=\underline{\frac{1-2\beta_{2}^{2}}{\beta_{2}^{2}}\Delta }+\frac{\beta_{2}^{2}}{c_{2,T}^{2}}\frac{\partial^{2}}{\partial t^{2}},\\ \; & \Omega_{i}=\underline{\left(2-\beta_{2}^{2}\right)\frac{\partial^{2}}{\partial x_{i}^{2}}+\frac{\partial^{2}}{\partial x_{j}^{2}}}-\frac{1}{c_{2,T}^{2}}\frac{\partial^{2}}{\partial t^{2}},\;\Delta =\frac{\partial^{2}}{\partial x_{i}^{2}}+\frac{\partial^{2}}{\partial x_{j}^{2}}, \end{array}$$

$j_{1,2}=0,1$ indicates the approximation order. In the case $\frac{\partial }{\partial x_{2}}=0$, operator $\Omega_{\mathrm{pl}}$ can be written in the form

$$\Omega_{\mathrm{pl}}=\frac{2}{1-\nu_{2}}\left(\frac{\partial^{2}}{\partial x_{1}^{2}}-\frac{\left(1-\nu_{2}^{2}\right)\rho_{2}}{E_{2}}\frac{\partial^{2}}{\partial t^{2}}\right).$$

Such an operator describes propagation of an extensional wave in a thin plate with free faces (see, e.g., [38]). In the problem under consideration, this operator becomes dominating only in the case $\mu_{2}\gg \mu$, which is not in the focus of the present investigation.

If $\xi_{l}^{\left(\pm 1\right)}=0$, the zero-order terms of (14) coincide with those obtained in [23,25]. The first order approximation ($j_{2}=0$) is in agreement with that in [23] except the terms with $j_{2}$ in the first two equations in (14), which referred to the first approximation in [23]. Apparently, this discrepancy is caused by the procedure of the EBCs derivation used by Rokhlin and Wang [23], which employs a transfer matrix for the vectors containing stresses and particle velocities.

If convenient, one can shift the vertical coordinate in domains $D_{1}$ and $D_{3}$ to calculate ${\hat{\sigma }}_{l3}^{\left(\pm 1\right)}$, ${\hat{u}}_{l}^{\left(\pm 1\right)}$ on the same surface in the global coordinate system, but this operation is not necessary.

In the zero order approximation ($j_{1}=j_{2}=0$), the EBCs are spring-type BCs

(16) $$\begin{array}{cccc} \; & {\hat{\sigma }}_{33}^{\left(1\right)}={\hat{\sigma }}_{33}^{\left(-1\right)}, & \; & {\hat{\sigma }}_{i3}^{\left(1\right)}={\hat{\sigma }}_{i3}^{\left(-1\right)},\\ \; & {\hat{u}}_{3}^{\left(1\right)}-{\hat{u}}_{3}^{\left(-1\right)}=\xi_{3}^{\mathrm{eff}}{\hat{\sigma }}_{33}^{\left(1\right)},\;\; & \; & {\hat{u}}_{i}^{\left(1\right)}-{\hat{u}}_{i}^{\left(-1\right)}=\xi_{i}^{\mathrm{eff}}{\hat{\sigma }}_{i3}^{\left(1\right)} \end{array}$$

with effective compliances

(17) $$\xi_{3}^{\mathrm{eff}}=\frac{h_{2}}{\lambda_{2}+2\mu_{2}}+\xi_{3}^{\left(1\right)}+\xi_{3}^{\left(-1\right)},\;\xi_{i}^{\mathrm{eff}}=\frac{h_{2}}{\mu_{2}}+\xi_{i}^{\left(1\right)}+\xi_{i}^{\left(-1\right)},$$

corresponding to effective stiffnesses $\varkappa_{1}^{\mathrm{eff}}={\left(\xi_{1}^{\mathrm{eff}}\right)}^{-1}$, $\varkappa_{3}^{\mathrm{eff}}={\left(\xi_{3}^{\mathrm{eff}}\right)}^{-1}$. Formulae (17) show that in the case of an imperfect contact the effective compliances are the sums of those of the film itself

(18) $$\xi_{3,0}^{\mathrm{eff}}=\frac{h_{2}}{\lambda_{2}+2\mu_{2}}=\frac{h_{2}\beta_{2}^{2}}{\mu_{2}},\;\xi_{i,0}^{\mathrm{eff}}=\frac{h_{2}}{\mu_{2}}$$

and those of the interfaces between the film and the external layers.

In the case of a symmetric laminate ($h_{1}=h_{3}=h$, $E_{1}=E_{3}$, $\nu_{1}=\nu_{3}$, $\rho_{1}=\rho_{3}$, $\xi_{l}^{\left(-1\right)}=\xi_{l}^{\left(1\right)}=\xi_{l}=\varkappa_{l}^{-1}$), it is convenient to shift the coordinate *y* locally in $D_{3}$ as $z=y-h_{2}/2$, and in $D_{1}$ as $z=y+h_{2}/2$. Then in the global coordinate system $\left(x_{1},x_{2},z\right)$ the surfaces of the waveguide are defined by z=±h and the interface by z=0. The boundary values ${\hat{\sigma }}_{l3}^{\left(\pm 1\right)}$, ${\hat{u}}_{l}^{\left(\pm 1\right)}$ mean the limits as z→±0 (we have a discontinuity here because of the film).

The problem can be separated into two independent ones: for the symmetric vibrations defined as

(19) $$\begin{array}{cc} \; & {\left.u_{i}^{\left(1\right)}\right|}_{z=-d}={\left.u_{i}^{\left(3\right)}\right|}_{z=d},\;{\left.u_{3}^{\left(1\right)}\right|}_{z=-d}=-{\left.u_{3}^{\left(3\right)}\right|}_{z=d},\\ \; & {\left.\sigma_{ll}^{\left(1\right)}\right|}_{z=-d}={\left.\sigma_{ll}^{\left(3\right)}\right|}_{z=d},\;\;{\left.\sigma_{ij}^{\left(1\right)}\right|}_{z=-d}={\left.\sigma_{ij}^{\left(3\right)}\right|}_{z=d},\;\;{\left.\sigma_{i3}^{\left(1\right)}\right|}_{z=-d}=-{\left.\sigma_{i3}^{\left(3\right)}\right|}_{z=d}, \end{array}$$

($d\in \left[0,h\right]$), and for the antisymmetric vibrations characterized by

(20) $$\begin{array}{cc} \; & {\left.u_{i}^{\left(1\right)}\right|}_{z=-d}=-{\left.u_{i}^{\left(3\right)}\right|}_{z=d},\;{\left.u_{3}^{\left(1\right)}\right|}_{z=-d}={\left.u_{3}^{\left(3\right)}\right|}_{z=d},\\ \; & {\left.\sigma_{ll}^{\left(1\right)}\right|}_{z=-d}=-{\left.\sigma_{ll}^{\left(3\right)}\right|}_{z=d},\;\;{\left.\sigma_{ij}^{\left(1\right)}\right|}_{z=-d}=-{\left.\sigma_{ij}^{\left(3\right)}\right|}_{z=d},\;\;{\left.\sigma_{i3}^{\left(1\right)}\right|}_{z=-d}={\left.\sigma_{i3}^{\left(3\right)}\right|}_{z=d}. \end{array}$$

It is sufficient to consider only one of two external layers, e.g., the upper one (the domain index is omitted below).

The symmetry properties imply

$$S_{33}={\hat{\sigma }}_{33},\;{\tilde{U}}_{i}={\hat{u}}_{i}-\xi_{i}{\hat{\sigma }}_{i3},\;P_{3}=2\xi_{3}{\hat{\sigma }}_{33},\;S_{i3}={\tilde{U}}_{3}=P_{i}=0$$

for the symmetric problem and

$$S_{i3}={\hat{\sigma }}_{i3},\;{\tilde{U}}_{3}={\hat{u}}_{3}-\xi_{3}{\hat{\sigma }}_{33},\;P_{i}=2\xi_{i}{\hat{\sigma }}_{i3},\;S_{33}={\tilde{U}}_{i}=P_{3}=0$$

for the antisymmetric one. According to these relations, two conditions in (14) are satisfied identically, and the other two give the EBCs for each case:

for the symmetric vibrations:

(21) $$\begin{array}{cc} \; & {\hat{\sigma }}_{i3}=-j_{1}h_{0}\left(1-2\beta_{2}^{2}\right)\frac{\partial {\hat{\sigma }}_{33}}{\partial x_{i}}-j_{2}h_{0}\mu_{2}\left[\Omega_{\mathrm{pl}}{\tilde{U}}_{i}+\underline{\left(3-4\beta_{2}^{2}\right)\frac{\partial^{2}{\tilde{U}}_{j}}{\partial x_{i}\partial x_{j}}}\right],\\ \; & {\hat{u}}_{3}=\frac{1}{2}\xi_{3}^{\mathrm{eff}}{\hat{\sigma }}_{33}-j_{1}h_{0}\left(1-2\beta_{2}^{2}\right)\left(\frac{\partial {\tilde{U}}_{i}}{\partial x_{i}}+\frac{\partial {\tilde{U}}_{j}}{\partial x_{j}}\right)-j_{2}\frac{h_{0}^{3}\beta_{2}^{2}}{3\mu_{2}}\Omega_{3}{\hat{\sigma }}_{33}; \end{array}$$

for the antisymmetric vibrations:

(22) $$\begin{array}{cc} \; & {\hat{\sigma }}_{33}=-j_{1}h_{0}\left(\frac{\partial {\hat{\sigma }}_{i3}}{\partial x_{i}}+\frac{\partial {\hat{\sigma }}_{j3}}{\partial x_{j}}\right)+j_{2}h_{0}\rho_{2}\frac{\partial^{2}{\tilde{U}}_{3}}{\partial t^{2}},\\ \; & {\hat{u}}_{i}=\frac{1}{2}\xi_{i}^{\mathrm{eff}}{\hat{\sigma }}_{i3}-j_{1}h_{0}\frac{\partial {\tilde{U}}_{3}}{\partial x_{i}}+j_{2}\frac{h_{0}^{3}}{3\mu_{2}}\left[\Omega_{i}{\hat{\sigma }}_{i3}+\underline{\left(1-\beta_{2}^{2}\right)\frac{\partial^{2}{\hat{\sigma }}_{j3}}{\partial x_{i}\partial x_{j}}}\right]. \end{array}$$

Here ${\hat{\sigma }}_{l3}$, ${\hat{u}}_{l}$ are boundary values on the lower surface of the upper layer.

The material parameters of soft films considered in this work (see Table 1) are related to the parameters of the external layers (aluminium) as $\rho_{2}/\rho_{1}∼1$, $\mu_{2}/\mu_{1}\ll 1$. An analysis of EBCs (21) and (22) taking into account the additional small parameter $\mu_{2}/\mu_{1}$ shows that the the underlined terms in (15), (21) and (22) are small. By omitting them we obtain the simplified EBCs used in Section 5.

### 2.3. Thickness Resonance Frequencies

Except the rare cases of backwards waves, the cut-off frequencies of LWs coincide with thickness resonance frequencies, which are eigenvalues of the problem analogous to that for LWs, but with wavenumber k=0 (see [38] for more details). In the laminate under consideration, these frequencies can be separated into two groups: thickness stretch resonance frequencies, for which the corresponding eigenforms satisfy conditions $u_{1}^{\left(q\right)}=0$, $\sigma_{13}^{\left(q\right)}=0$, and thickness shear resonance frequencies with $u_{3}^{\left(q\right)}=0$, $\sigma_{33}^{\left(q\right)}=0$.

For the antisymmetric vibrations, the thickness stretch resonance frequencies are defined as $f_{n,\mathrm{st}}^{a}=\omega_{n,\mathrm{st}}^{a}/2\pi$, where $\omega_{n,\mathrm{st}}^{a}$ (n=1,2,…) are ω-roots of equation

(23) $$\sin\frac{\omega h}{c_{1,L}}\left(\cos\frac{\omega h_{2}}{2c_{2,L}}-\frac{\sqrt{\mu_{2}\rho_{2}}}{\varkappa_{3}\beta_{2}}\omega \sin\frac{\omega h_{2}}{2c_{2,L}}\right)+\frac{\beta_{1}\sqrt{\mu_{2}\rho_{2}}}{\beta_{2}\sqrt{\mu_{1}\rho_{1}}}\sin\frac{\omega h_{2}}{2c_{2,L}}\cos\frac{\omega h}{c_{1,L}}=0.$$

In the case of a soft interlayer, we have $\mu_{2}/\mu_{1}\ll 1$, so the roots of Equation (23) allow additional separation in two groups, approximately defined by equations

(24) $$\sin\frac{\omega h}{c_{1,L}}=0$$

or

(25) $$\cos\frac{\omega h_{2}}{2c_{2,L}}-\frac{\sqrt{\mu_{2}\rho_{2}}}{\varkappa_{3}\beta_{2}}\omega \sin\frac{\omega h_{2}}{2c_{2,L}}=0.$$

The roots of Equations (24) and (25) are thickness stretch resonance frequencies of the external layers and the film with SBCs on its surfaces, respectively. In the case of a thin film, the thickness resonances are extremely high-frequency ones. But in the case $\mu_{2}/\mu_{1}\ll 1$, which is considered here, these resonances can arise at the relatively low frequencies. If $\varkappa_{3}\to \infty$, the lowest thickness stretch resonance frequency of the film is defined by the lowest root of equation $\cos\frac{\omega h_{2}}{2c_{2,L}}=0$. If $\varkappa_{3}\to 0$, this frequency can be approximately described by two-term asymptotic approximation:

(26) $$f_{\mathrm{fl},\mathrm{st}}^{a}\approx \left\{\begin{array}{c} \frac{1}{2\pi }\sqrt{\frac{2\varkappa_{3}}{\rho_{2}h_{2}\left(1+\frac{\varkappa_{3}h_{2}\beta_{2}^{2}}{6\mu_{2}}\right)}}\;\;\mathrm{if}\;\varkappa_{3}\to 0,\\ \frac{c_{2,L}}{2h_{2}}\;\;\mathrm{if}\;\varkappa_{3}\to \infty . \end{array}\right.$$

For the 50 μm-thick two-sided epoxy tape from Table 1, $f_{\mathrm{fl},\mathrm{st}}^{a}=10.7$ MHz at $\varkappa_{3}=\infty$ and $f_{\mathrm{fl},\mathrm{st}}^{a}<3$ MHz if $\varkappa_{3}<9\;\mathrm{GPa}/\mathrm{mm}$. The number *n* of the frequency (26) in the series $f_{n,\mathrm{st}}^{a}$ depends on the relation between parameters of the external layers and the film, including the interface stiffnesses. In the case when the frequency (26) coincides with some of the root of Equation (24), so-called “repulsion effect” arises, so we do not have multiply root in such a situation.

The antisymmetric thickness shear resonance frequencies are defined as $f_{n,\mathrm{sh}}^{a}=\omega_{n,\mathrm{sh}}^{a}/2\pi$, where $\omega_{n,\mathrm{sh}}^{a}$ are ω-roots of equation

(27) $$\sin\frac{\omega h}{c_{1,T}}\left(\sin\frac{\omega h_{2}}{2c_{2,T}}+\frac{\sqrt{\mu_{2}\rho_{2}}}{\varkappa_{1}}\omega \cos\frac{\omega h_{2}}{2c_{2,T}}\right)-\frac{\sqrt{\mu_{2}\rho_{2}}}{\sqrt{\mu_{1}\rho_{1}}}\cos\frac{\omega h_{2}}{2c_{2,T}}\cos\frac{\omega h}{c_{1,T}}=0,$$

which can be separated analogously to Equation (23) if $\mu_{2}/\mu_{1}\ll 1$, except the case of the lowest thickness shear resonance frequency $f_{1,\mathrm{sh}}^{a}$. The latter cannot be observed in the film considered separately, and is characteristic only for three-layered waveguides. In a strongly inhomogeneous waveguide, the part of the dispersion curve starting from this frequency comes to be in the long-wave range in respect to the external layers (i.e., L≫h, where *L* is the characteristic wavelength). This case is thoroughly studied in [40], where the two-mode asymptotic polynomial expansions of the Rayleigh-Lamb dispersion relation approximating both the fundamental antisymmetric wave and the first high order wave can be found. In the laminate considered in the present paper, this effect can be obtained if $\mu_{2}$ or $\varkappa_{1}$ is sufficiently small. The antisymmetric thickness shear resonance frequencies of the film are high and not of interest for the present investigation.

The thickness stretch resonance frequencies for the symmetric vibrations are defined as $f_{n,\mathrm{st}}^{s}=\omega_{n,\mathrm{st}}^{s}/2\pi$, where $\omega_{n,\mathrm{st}}^{s}$ are ω-roots of equation

(28) $$\sin\frac{\omega h}{c_{1,L}}\left(\sin\frac{\omega h_{2}}{2c_{2,L}}+\frac{\sqrt{\mu_{2}\rho_{2}}}{\varkappa_{3}\beta_{2}}\omega \cos\frac{\omega h_{2}}{2c_{2,L}}\right)-\frac{\beta_{1}\sqrt{\mu_{2}\rho_{2}}}{\beta_{2}\sqrt{\mu_{1}\rho_{1}}}\cos\frac{\omega h_{2}}{2c_{2,L}}\cos\frac{\omega h}{c_{1,L}}=0.$$

The properties of the roots of Equation (28) are analogous to the ones of Equation (27). In particular, the lowest thickness stretch resonance frequency $f_{1,\mathrm{st}}^{s}$ is also of the type, that is characteristic only for three-layered waveguides and comes to be in low-frequency range in respect to the external layers, if $\mu_{2}/\beta_{2}$ or $\varkappa_{3}$ is sufficiently small. The thickness stretch resonance frequencies of the film are very high in this case.

The symmetric shear resonance frequencies are of more interest for the present investigation. They are defined as $f_{n,\mathrm{sh}}^{s}=\omega_{n,\mathrm{sh}}^{s}/2\pi$, where $\omega_{n,\mathrm{sh}}^{s}$ are ω-roots of equation

(29) $$\sin\frac{\omega h}{c_{1,T}}\left(\cos\frac{\omega h_{2}}{2c_{2,T}}-\frac{\sqrt{\mu_{2}\rho_{2}}}{\varkappa_{1}}\omega \sin\frac{\omega h_{2}}{2c_{2,T}}\right)+\frac{\sqrt{\mu_{2}\rho_{2}}}{\sqrt{\mu_{1}\rho_{1}}}\sin\frac{\omega h_{2}}{2c_{2,T}}\cos\frac{\omega h}{c_{1,T}}=0.$$

As $\mu_{2}/\mu_{1}\ll 1$, the roots of Equation (29) can be separated into two groups, approximately defined by equations

(30) $$\sin\frac{\omega h}{c_{1,T}}=0$$

or

(31) $$\cos\frac{\omega h_{2}}{2c_{2,T}}-\frac{\sqrt{\mu_{2}\rho_{2}}}{\varkappa_{1}}\omega \sin\frac{\omega h_{2}}{2c_{2,T}}=0.$$

As for Equations (24) and (25), we have here the thickness shear resonance frequencies of the external layers (Equation (30)) and those of the film with SBCs on its surfaces (Equation (31)), acting, in contrast with (25), in the tangential direction. If $\varkappa_{1}\to \infty$, the lowest thickness shear resonance frequency of the film is defined by the lowest root of equation $\cos\frac{\omega h_{2}}{2c_{2,T}}=0$. As $\varkappa_{1}\to 0$, two-term asymptotic approximation can be derived, so we have

(32) $$f_{\mathrm{fl},\mathrm{sh}}^{s}\approx \left\{\begin{array}{c} \frac{1}{2\pi }\sqrt{\frac{2\varkappa_{1}}{\rho_{2}h_{2}\left(1+\frac{\varkappa_{1}h_{2}}{6\mu_{2}}\right)}}\;\;\mathrm{if}\;\varkappa_{1}\to 0,\\ \frac{c_{2,T}}{2h_{2}}\;\;\mathrm{if}\;\varkappa_{1}\to \infty . \end{array}\right.$$

For the 50 μm-thick two-sided epoxy tape with the material properties given in Table 1, $f_{\mathrm{fl},\mathrm{sh}}^{s}<3$ MHz if $\varkappa_{1}<15$ GPa/mm.

In the narrow frequency ranges of the thickness resonance frequencies of the film the vibrations of the interlayer are of the long-wave, high-frequency type [38]. In this case, the assumptions made by integration of the system (10) are not valid, so the EBCs constructed above are not applicable in these regions. In [38], the procedure of asymptotic analysis specified for the long-wave, high-frequency vibrations, is developed, which could be also applied in the case under consideration. In the present work, we restrict ourselves to long-wave, low-frequency EBCs, constructed above.

## 3. Properties of Lamb Waves in Laminates with Soft Interlayer

### 3.1. Main Properties of Dispersion Curves and Vibration Forms

**Figure 2:**
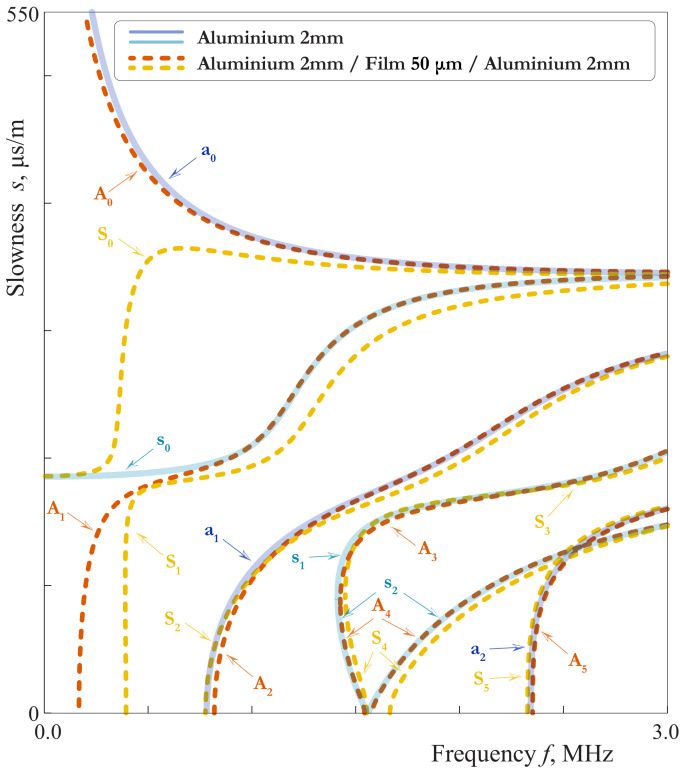
Slownesses of LWs propagating in 4.05 mm thickness plate (2 mm aluminium/50 μm film/2 mm aluminium) and 2 mm thickness aluminium plate.

In this paper, slowness curves (SCs) s=k(ω)/ω are investigated since SCs for various GWs could be easier distinguished compared to wavenumbers *k* or phase velocities ω/k(ω). Some SCs for LWs in the three-layered waveguide with soft interlayer might be somehow similar to the SCs for pure upper or lower waveguides analogous to [19,26]. To investigate this effect more detailed, it is natural to compare guiding properties of the symmetric laminate with a soft thin mid-layer and a homogeneous waveguide, which is the first layer of the laminate. Figure 2 demonstrates SCs of 4.05 mm thickness plate (2 mm aluminium/50 μm film/2 mm aluminium) and 2 mm thickness aluminium plate. Hereinafter, SCs of symmetric and antisymmetric elastic waves are shown using lines of different colours. To distinguish SCs for the two considered waveguides, capital letters are used for the laminate ($A_{0}$, $S_{0}$, *…*), while lower-case letters denote GWs propagating in the layer ($a_{0}$, $s_{0}$, *…*). It is observed that the SCs of different GWs propagating in the laminate and in the layer almost coincide in wide frequency ranges. For example, these are $a_{0}$ and $A_{0}$ modes if f>0.8 MHz; $s_{0}$ for the layer and $A_{1}$ for the laminate if f>0.7 MHz; $a_{1}$, $s_{1}$ for the layer and $S_{2}$, $A_{2}$ and for the laminate for all the frequencies higher than their cut-off frequencies. The largest discrepancy between SCs for two considered waveguides occurs for first GWs.

**Figure 3:**
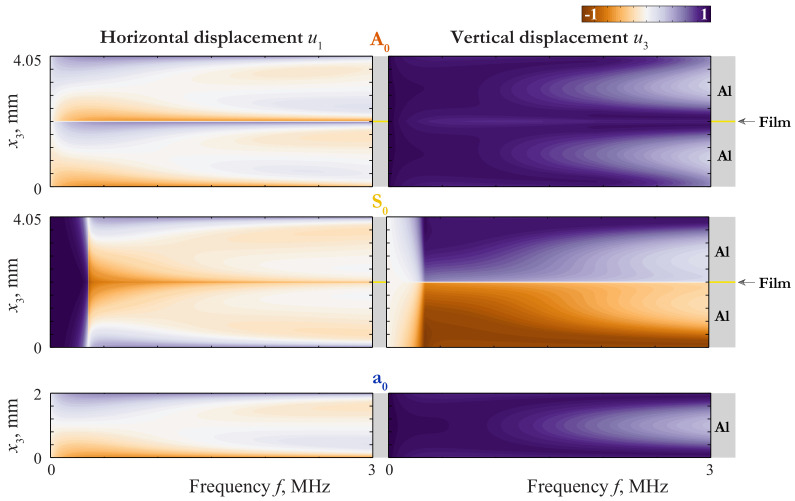
Displacement distribution $u_{k}\left(x_{3},f\right)$ of LWs $A_{0}$ and $S_{0}$ propagating in 4.05 mm thickness plate (2 mm aluminium/50 μm film/2 mm aluminium) and LW $a_{0}$ propagating in 2 mm thickness aluminium plate.

**Figure 4:**
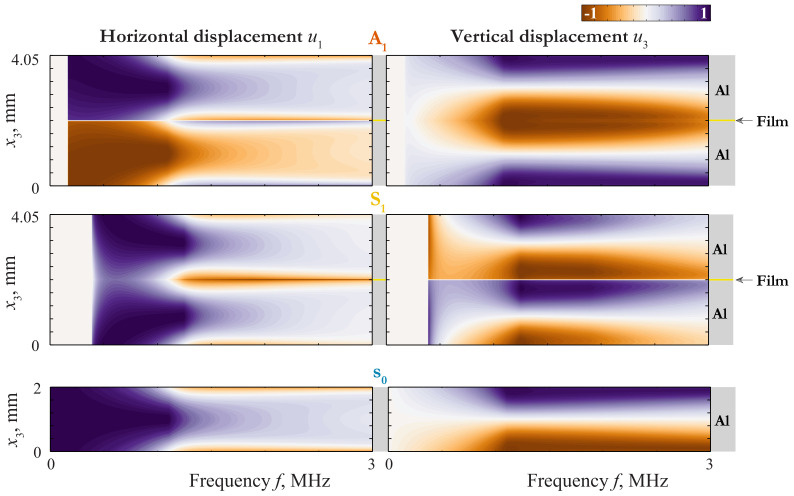
Displacement distribution $u_{k}\left(x_{3},f\right)$ of LWs $A_{1}$ and $S_{1}$ propagating in 4.05 mm thickness plate (2 mm aluminium/50 μm film/2 mm aluminium) and LW $s_{0}$ propagating in 2 mm thickness aluminium plate.

**Figure 5:**
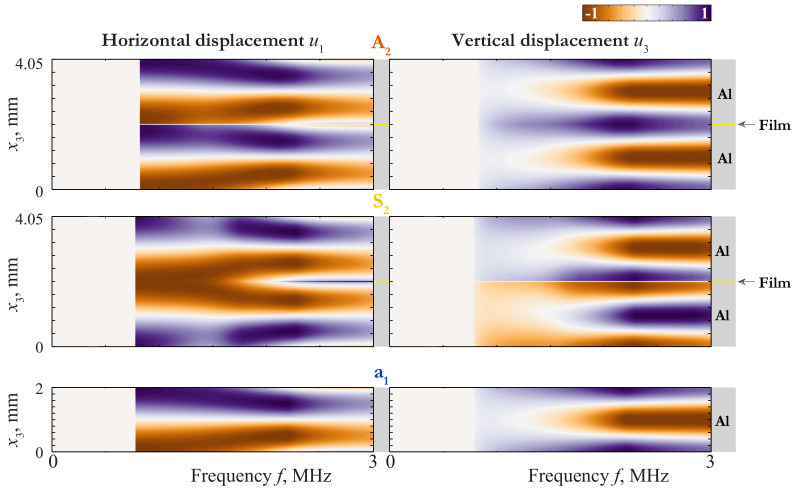
Displacement distribution $u_{k}\left(x_{3},f\right)$ of LWs $A_{2}$ and $S_{2}$ propagating in 4.05 mm thickness plate (2 mm aluminium/50 μm film/2 mm aluminium) and LW $a_{1}$ propagating in 2 mm thickness aluminium plate.

An insight into the nature of such a coincidence can be given via the consideration of the wave-fields corresponding to these GWs. The displacement distribution of LWs propagating in 4.05 mm thickness plate (2 mm aluminium/50 μm film/2 mm aluminium) and in 2 mm thickness aluminium plate are depicted in Figure 3, Figure 4 and Figure 5, where the variation of horizontal $u_{1}\left(x_{3},f\right)$ and vertical $u_{3}\left(x_{3},f\right)$ components of the displacement vector of the first Lamb waves (LWs) are shown as contour plots.

The employment of EBCs (21) and (22) provides better understanding of peculiar properties of LWs revealed in numerical investigation. Let us write down the boundary conditions for the upper layer with the zero-order approximation of EBCs ($j_{1}=j_{2}=0$): for the symmetric vibrations:

(33) $$\begin{array}{cc} \; & {\left.\sigma_{13}\right|}_{z=h}={\left.\sigma_{33}\right|}_{z=h}=0,\\ \; & {\left.\sigma_{13}\right|}_{z=0}=0,\;{\left.\sigma_{33}\right|}_{z=0}=\frac{2\mu_{2}}{h_{2}\beta_{2}^{2}}{\left.u_{3}\right|}_{z=0}; \end{array}$$

for the antisymmetric vibrations:

(34) $$\begin{array}{cc} \; & {\left.\sigma_{33}\right|}_{z=h}={\left.\sigma_{13}\right|}_{z=h}=0,\\ \; & {\left.\sigma_{33}\right|}_{z=0}=0,\;{\left.\sigma_{13}\right|}_{z=0}=\frac{2\mu_{2}}{h_{2}}{\left.u_{1}\right|}_{z=0}. \end{array}$$

After solving the problem for the upper layer, one can construct the wave-field in the lower one by continuation of the solution according to (19) or (20). From BCs (33) and (34), one can easily see that the problem is reduced to the statement for a single layer with stress-free BCs on the top surface and elastically constrained the bottom one. Notice also, that the elastic constraint acts only in the normal direction to the surface in the symmetric case (33). On the contrary, in the antisymmetric case (34) we have the elastic constraint only in the tangential direction. To analyze the influence of this constraint, one must take into account the properties of LWs in a single layer.

On the basis of the asymptotic analysis carried out in [38], the relations for the first LWs can be obtained. Thus,

for mode $s_{0}$:

(35) $$\sigma_{13}=E_{1}\epsilon^{4}{\bar{\sigma }}_{13},\;\sigma_{33}=E_{1}\epsilon^{3}{\bar{\sigma }}_{33},\;u_{3}=\frac{h}{2}\epsilon {\bar{u}}_{3},\;u_{1}=\frac{h}{2}{\bar{u}}_{1},\;\;{\bar{\sigma }}_{13}∼{\bar{\sigma }}_{33}∼{\bar{u}}_{3}∼{\bar{u}}_{1};$$

for mode $a_{0}$:

(36) $$\sigma_{33}=E_{1}\epsilon^{4}{\bar{\sigma }}_{33},\;\sigma_{13}=E_{1}\epsilon^{3}{\bar{\sigma }}_{13},\;u_{1}=\frac{h}{2}\epsilon {\bar{u}}_{1},\;u_{3}=\frac{h}{2}{\bar{u}}_{3},\;\;{\bar{\sigma }}_{33}∼{\bar{\sigma }}_{13}∼{\bar{u}}_{1}∼{\bar{u}}_{3};$$

for modes $s_{1}$, $a_{1},\ldots$:

(37) $$\sigma_{13}=E_{1}{\bar{\sigma }}_{13},\;\sigma_{33}=E_{1}{\bar{\sigma }}_{33},\;u_{3}=\frac{h}{2}{\bar{u}}_{1},\;u_{1}=\frac{h}{2}{\bar{u}}_{3},\;\;{\bar{\sigma }}_{13}∼{\bar{\sigma }}_{33}∼{\bar{u}}_{3}∼{\bar{u}}_{1}.$$

Here ϵ=πh/L, where *L* is the characteristic wavelength. For the mode $s_{0}$, this wavelength can be roughly estimated as $L∼c_{\mathrm{pl}}/f$, where $c_{1,\mathrm{pl}}=\sqrt{E_{1}/\left(1-\nu_{1}^{2}\right)\rho_{1}}$ and *f* is the frequency. For the other modes of a single layer, $L∼c_{1,T}/f$. All the modes of a single layer satisfy three BCs out of four BCs in (33) and (34). Let us investigate the last BC, considering the antisymmetric mode of the laminate as a couple of antisymmetric modes $a_{0}$. Introducing (36) in (34), we have

(38) $$\epsilon^{2}{\left.{\bar{\sigma }}_{13}\right|}_{z=0}=\frac{h\mu_{2}}{h_{2}E_{1}}{\left.{\bar{u}}_{1}\right|}_{z=0}\to \left\{\begin{array}{c} {\left.{\bar{u}}_{1}\right|}_{z=0}=0,\;\epsilon \to 0;\\ {\left.{\bar{\sigma }}_{13}\right|}_{z=0}=K{\left.{\bar{u}}_{1}\right|}_{z=0},\;\epsilon ∼{\left(\frac{h\mu_{2}}{h_{2}E_{1}}\right)}^{1/2};\\ {\left.{\bar{\sigma }}_{13}\right|}_{z=0}=O\left(\frac{h\mu_{2}}{h_{2}E_{1}}\right){\left.{\bar{u}}_{1}\right|}_{z=0},\;\epsilon ∼1. \end{array}\right.$$

In the numerical example under consideration, the shear interlayer parameter is

(39) $$\frac{h\mu_{2}}{h_{2}E_{1}}=0.1\ll 1.$$

Expressing ϵ through *f* ($\epsilon =\pi hf/c_{1,T}$), one can estimate the transition frequency, corresponding to $\epsilon ∼{\left(\frac{h\mu_{2}}{h_{2}E_{1}}\right)}^{1/2}$: $f_{\mathrm{trans}}=0.16$ MHz. One can see from (38) that the laminate behaves itself approximately as an antisymmetric couple of antisymmetric modes $a_{0}$ at $f\gg f_{\mathrm{trans}}$, as a single layer of the thickness 2h=4mm at $f\ll f_{\mathrm{trans}}$, and in the vicinity of $f_{\mathrm{trans}}$ as an antisymmetric couple of modes in a layer of the thickness h=2mm with a strong elastic constraint at the bottom surface. In the last two cases, the BCs for the upper layer are essentially asymmetric, so the waveform must be different from that of $a_{0}$. All these proprieties can be seen by mode $A_{0}$ in Figure 3.

The other antisymmetric modes are not fundamental, so the using of (35) with ϵ→0 has no sense for them. Let us consider the antisymmetric mode of the laminate as a couple of symmetric modes $s_{0}$. Introducing (35) in (34), we have

(40) $$\epsilon^{4}{\left.{\bar{\sigma }}_{13}\right|}_{z=0}=\frac{h\mu_{2}}{h_{2}E_{1}}{\left.{\bar{u}}_{1}\right|}_{z=0}\to \left\{\begin{array}{c} {\left.{\bar{\sigma }}_{13}\right|}_{z=0}=K{\left.{\bar{u}}_{1}\right|}_{z=0},\;\epsilon ∼{\left(\frac{h\mu_{2}}{h_{2}E_{1}}\right)}^{1/4};\\ {\left.{\bar{\sigma }}_{13}\right|}_{z=0}=O\left(\frac{h\mu_{2}}{h_{2}E_{1}}\right){\left.{\bar{u}}_{1}\right|}_{z=0},\;\epsilon ∼1. \end{array}\right.$$

In this case $\epsilon =\pi hf/c_{1,\mathrm{pl}}$ and $f_{\mathrm{trans}}=0.48$ MHz. The behaviour defined by (40) can be observed by mode $A_{1}$ in Figure 2 and Figure 4.

Now let us consider the symmetric mode of the laminate as a couple of symmetric modes $s_{0}$. Introducing (35) in (33), we have

(41) $$\epsilon^{2}{\left.{\bar{\sigma }}_{33}\right|}_{z=0}=\frac{h\mu_{2}}{h_{2}E_{1}\beta_{2}^{2}}{\left.{\bar{u}}_{3}\right|}_{z=0}\to \left\{\begin{array}{c} {\left.{\bar{u}}_{3}\right|}_{z=0}=0,\;\epsilon \to 0;\\ {\left.{\bar{\sigma }}_{33}\right|}_{z=0}=K{\left.{\bar{u}}_{3}\right|}_{z=0},\;\epsilon ∼{\left(\frac{h\mu_{2}}{h_{2}E_{1}\beta_{2}^{2}}\right)}^{1/2};\\ {\left.{\bar{\sigma }}_{33}\right|}_{z=0}=O\left(\frac{h\mu_{2}}{h_{2}E_{1}\beta_{2}^{2}}\right){\left.{\bar{u}}_{3}\right|}_{z=0},\;\epsilon ∼1, \end{array}\right.$$

and $f_{\mathrm{trans}}=0.67$ MHz. Here the stretch interlayer parameter

(42) $$\frac{h\mu_{2}}{h_{2}E_{1}\beta_{2}^{2}}=0.6$$

is not so small as the shear one given by (39). Consequently, the influence of the elastic constraint on symmetric modes of the laminate is stronger than that on the antisymmetric ones. For the symmetric mode of the laminate considered as a couple of antisymmetric modes $a_{0}$ we introduce (36) in (34) and obtain

(43) $$\epsilon^{4}{\left.{\bar{\sigma }}_{33}\right|}_{z=0}=\frac{h\mu_{2}}{h_{2}E_{1}\beta_{2}^{2}}{\left.{\bar{u}}_{3}\right|}_{z=0}\to \left\{\begin{array}{c} {\left.{\bar{\sigma }}_{33}\right|}_{z=0}=K{\left.{\bar{u}}_{3}\right|}_{z=0},\;\epsilon ∼{\left(\frac{h\mu_{2}}{h_{2}E_{1}\beta_{2}^{2}}\right)}^{1/4};\\ {\left.{\bar{\sigma }}_{33}\right|}_{z=0}=O\left(\frac{h\mu_{2}}{h_{2}E_{1}\beta_{2}^{2}}\right){\left.{\bar{u}}_{3}\right|}_{z=0},\;\epsilon ∼1 \end{array}\right.$$

with $\epsilon =\pi hf/c_{1,T}$ and $f_{\mathrm{trans}}=0.44\;\mathrm{MHz}$. Here the behaviour of the laminate is complicated by the repulsion effect, because of which the mode $S_{0}$ begins as a one defined by (41) and transforms to one defined by (43) at f>0.5MHz, and visa versa for $S_{1}$. As $f\ll f_{\mathrm{trans}}$, the mode $S_{0}$ behaves as mode $s_{0}$ for a single layer of the thickness 2h=4mm. The slowness of $s_{0}$ in the long-wave range do not depend on the thickness, so the transition from the first line of (41) to the third means that the coincidence between the SC of $s_{0}$ and that of the laminate becomes worse at $f\gg f_{\mathrm{trans}}=0.67\;\mathrm{MHz}$. With taking into account the repulsion effect, one can see the behaviour defined by (41) and (43) by modes $S_{0}$ and $S_{1}$ in Figure 2, Figure 3 and Figure 4.

**Figure 6:**
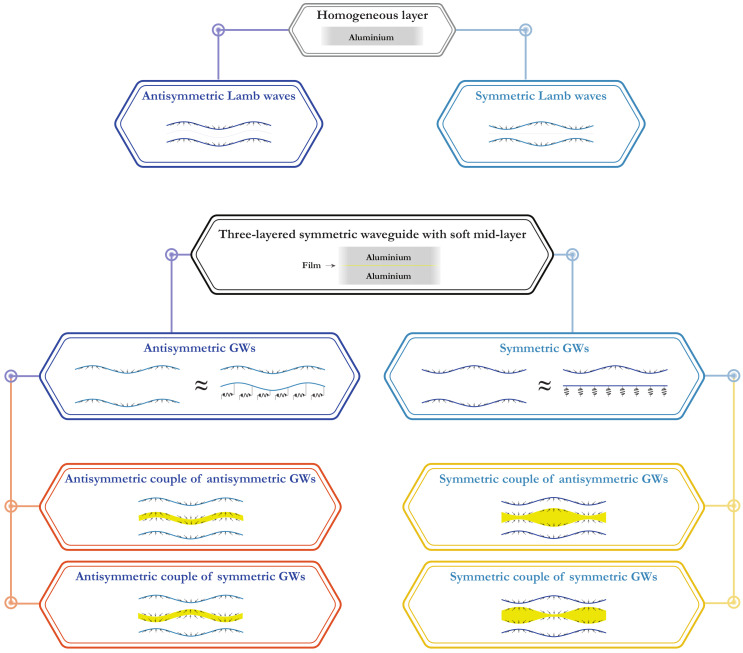
Classification of GWs propagating in homogeneous elastic waveguide and symmetric three-layered waveguide with thin soft mid-layer.

Introducing (37) in (33) and (34), we obtain the third lines in (41) and (38), respectively. Thus, all the next modes can be approximately considered as couples of modes of the single 2mm-thickness aluminium layer (see an example of this behaviour in Figure 5). The classification of possible variants is presented in Figure 6. Notice, that the accuracy of this scheme depends on the values of parameters (39) and (42). The asymptotic behaviour (37) is not applicable in the vicinities of the thickness resonance frequencies (see [38]). These vicinities are rather narrow, so they are not considered in the present work.

### 3.2. Influence of the Mechanical Properties of Interlayer

**Figure 7:**
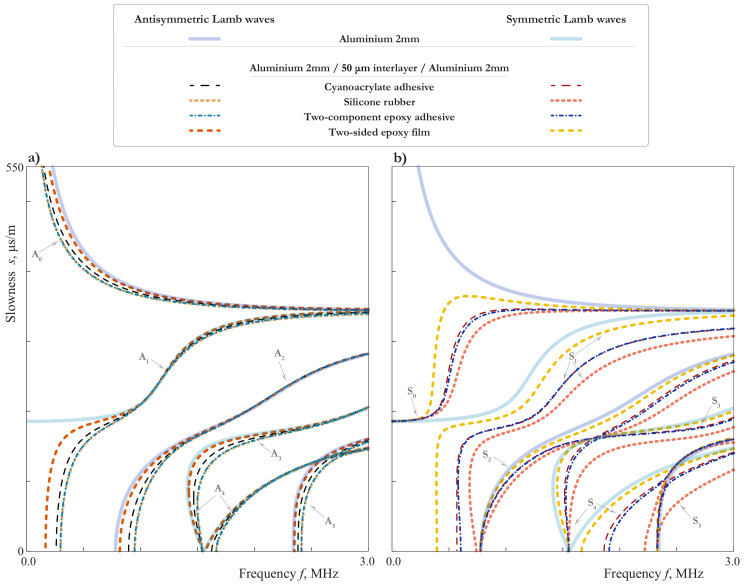
Slownesses of antisymmetric (**a**) and symmetric (**b**) LWs propagating in 4.05 mm thickness plate (2 mm aluminium/50 μm interlayer/2 mm aluminium) for four materials: two-sided epoxy tape (dashed thick lines), two-component epoxy adhesive (dash-dotted lines), cyanoacrylate adhesive (dashed thin lines), silicone rubber (thick dotted lines).

Let us investigate the influence of the mechanical properties of the soft thin interlayer on the characteristics of LWs propagating such as the considered symmetric laminate. Figure 7 exhibits SCs for LWs propagating in 4.05 mm thickness plate (2 mm aluminium/50 μm interlayer/2 mm aluminium) with perfect contact BCs at the interfaces, where properties of four various materials listed in Table 1 are employed to simulate thin soft interlayer: two-sided epoxy tape (dashed thick lines), two-component epoxy adhesive (dash-dotted lines), cyanoacrylate adhesive (dashed thin lines), silicone rubber (thick solid lines). The Young’s moduli of adhesives vary in a relatively narrow range, whereas Poisson ratios belong to a wide range including most of the adhesives (0.35≤ν≤0.48).

One can see that most of the SCs are dissimilar for the considered materials, although there are frequency ranges, where SCs for all the materials coincide (1–1.3 MHz for $A_{1}$, 1.5–3 MHz for $A_{2}$). All these peculiarities can be explained based on the analysis, presented above. For example, the close values of the tangential effective stiffness ($\varkappa_{1}^{\mathrm{eff}}=20.4\;\mathrm{GPa}/\mathrm{mm}$ for two-component epoxy adhesive, $\varkappa_{1}^{\mathrm{eff}}=20.9\;\mathrm{GPa}/\mathrm{mm}$ for silicone rubber) explains the fact, that all the antisymmetric modes for these two materials coincide. The SCs of symmetric modes for two-component epoxy adhesive ($\varkappa_{3}^{\mathrm{eff}}=88.3\;\mathrm{GPa}/\mathrm{mm}$) and cyanoacrylate adhesive ($\varkappa_{3}^{\mathrm{eff}}=72.9\;\mathrm{GPa}/\mathrm{mm}$) lay close together in all the frequency range up to 3 MHz. For the silicone rubber, the value of the tangential effective stiffness become extremely large ($\varkappa_{3}^{\mathrm{eff}}=544.6\;\mathrm{GPa}/\mathrm{mm}$) because of the small value of $\beta_{2}$ for such a Poisson’s ratio, and the latter explains the peculiar behaviour of symmetric modes. Thus, it can be concluded that the dissimilar material properties of the thin soft interlayer lead to distinguishable dissimilar SCs.

The comparison of SCs for the laminate and the aluminium sublayer shows that the growing of the Young modulus $E_{2}$ influences the symmetric couples of sublayer modes more than the antisymmetric ones. This fact could be explained on the basis of the scheme in Figure 6, since it is obviously easier to bend a thin film than to stretch it in the transverse direction.

### 3.3. Influence of the Thickness of Interlayer

**Figure 8:**
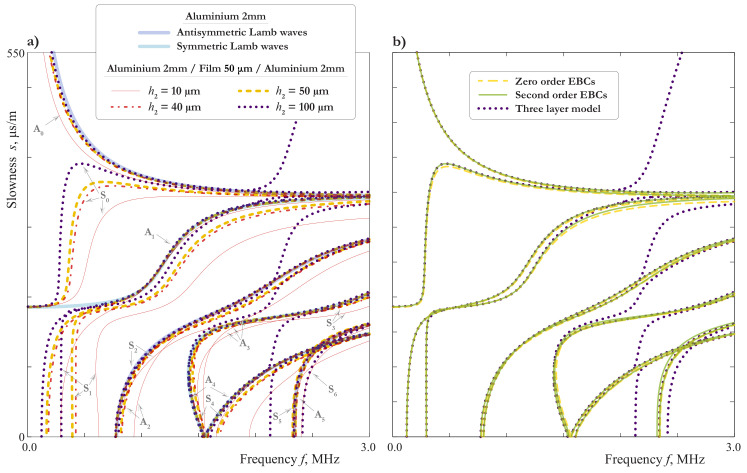
SCs of LWs propagating in the laminate (2 mm aluminium/$h_{2}$ thickness film/2 mm aluminium) at $h_{2}=10,40,50,100\;\mu$m (**a**) and in 4.1 mm thickness plate ($h_{2}=100\;\mu$m) calculated using EBCs (21) and (22) of zero order (thin dashed lines), second order (solid lines), three layer model (thick dashed lines) (**b**).

Figure 8a demonstrates SCs for four different values of $h_{2}$ LWs and illustrate the influence of the soft interlayer thickness on the SCs. A discrepancy distinguished by eye can be observed even for two similar thicknesses $h_{2}=40$ μm (dash-dotted lines) and $h_{2}=50$ μm (dashed lines). One can also see, that the SCs move close to those of an aluminium sublayer, when the thickness of the film grows, except the narrow frequency ranges near the cut-offs of the film. This effect is in agreement with formulae (18) and the analysis performed in Section 2.3 and Section 3.1.

In Figure 8b, the SCs for LWs calculated using EBCs (21) and (22) are compared with those computed using the exact three layer model. Up to 1.25 MHz, on can see no difference between SCs obtained with the use of the SBCs (zero order EBCs, $j_{1}=j_{2}=0$) and by the exact three-layer model, except the region around the “turn”of mode $S_{0}$. For the second order EBCs ($j_{1}=j_{2}=1$), one can see a good agreement everywhere except the frequency range around 2.2 MHz, where the EBCs do not describe an additional mode. This is an example of the effect mentioned in the end of Section 2.3 and related to the thickness resonance frequencies of the film. Indeed, for the two-sided epoxy tape with the parameters listed in the Table 1 and $\varkappa_{1}=\infty$, the formula (32) gives $f_{\mathrm{fl},\mathrm{sh}}^{s}=2.2$ MHz for the 100μm-film. For the 50μm-film, we have $f_{\mathrm{fl},\mathrm{sh}}^{s}=4.4$ MHz. In this case, the agreement between the three layer model and one with second order EBCs (21) and (22) is very good up to 3 MHz.

### 3.4. Influence of the Adhesive Bonding or Imperfect Contact

**Figure 9:**
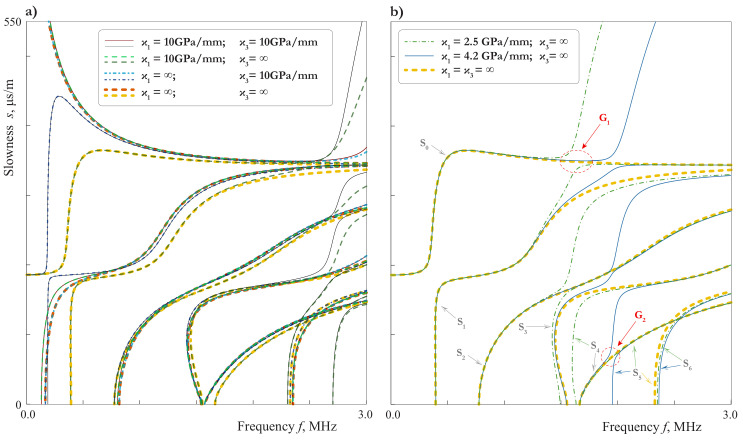
SCs of symmetric LWs propagating in 4.05 mm thickness plate (2 mm aluminium/50 μm film/2 mm aluminium) with the imperfect contact for four different combinations of normal and tangential stiffnesses $\varkappa_{i}$ (**a**) and symmetric LWs for two different values of the interface tangential stiffness if $\varkappa_{3}=\infty$ (**b**).

The condition of the perfect contact is an idealization, which, from the practical point of view, can be considered only as an approximation. The estimation of the applicability of such an approximation is not a trivial problem. The possible way to solve it may be found with the use of mathematical modeling, in which the possible contact degradation is taken into account via the SBCs (3). In Figure 9, the SCs for various combinations of $\varkappa_{1}$ and $\varkappa_{3}$ are presented. Comparing this figure with Figure 7 and Figure 8a, one can see that it is hard to distinguish between the effects of the thickness, the material properties or the interface stiffnesses variation, unless the thickness resonance frequency of the film comes to be in the considered frequency domain, as in Figure 8a for the 100μm-thick film. This is explained by Figure 8b, which show that the principal behaviour of SCs can be described by the model with zero order EBCs, in which the parameters of the film are presented only through combinations (17). Still, the extraction of all the film-related parameters including the interface stiffnesses from dispersion properties of LWs is possible, when the experimental data meet certain requirements, which are investigated in Section 3.5.

In the case of weakened interfaces with large values of $\xi_{l}=\varkappa_{l}^{-1}$, the numerical calculation of dispersion curves the 50μm-film reveals the effect of sharp increase of slowness analogous to that shown in Figure 8. It can be seen from Figure 9a, that this effect is most likely to be observed by symmetric modes, when the interface is weakened in the tangential direction.

**Figure 10:**
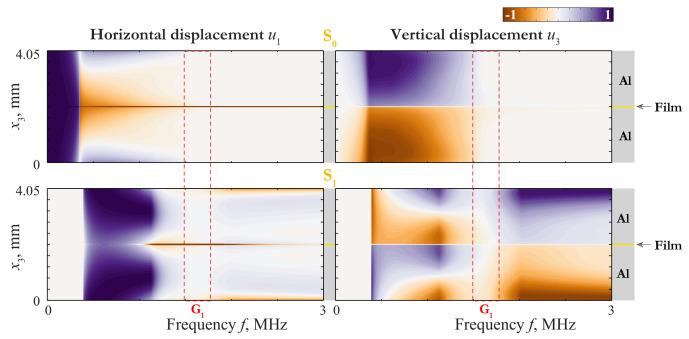
Displacement distribution $u_{k}\left(x_{3},f\right)$ of SLWs propagating in 4.05 mm thickness plate (2 mm aluminium/50 μm film/2 mm aluminium) with imperfect contact ($\kappa_{1}=2.5$ GPa/mm, $\kappa_{3}=\infty$).

**Figure 11:**
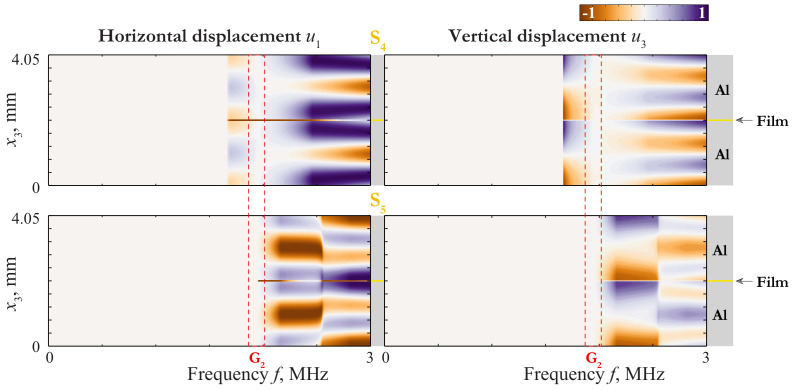
Displacement distribution $u_{k}\left(x_{3},f\right)$ of SLWs propagating in 4.05 mm thickness plate (2 mm aluminium/50 μm film/2 mm aluminium) with imperfect contact ($\kappa_{1}=4.2$ GPa/mm, $\kappa_{3}=\infty$).

Two examples of SCs for symmetric modes in the case of continuous vertical displacements, i.e., $\varkappa_{3}=\infty$, are presented in Figure 9b for $\varkappa_{1}=2.5\;\mathrm{GPa}/\mathrm{mm}$ and $\varkappa_{1}=4.2\;\mathrm{GPa}/\mathrm{mm}$. The investigation of vibration forms shows that in the vicinity of the frequency (32) they are characterized by “trapping”of the energy by the film, so the external layers nearly cease to move at all. Since the measurements of the wave-field are usually made on the outer surfaces of the laminate, this effect can manifest itself only as gaps in the experimentally acquired dispersion curves of symmetric modes. For example, for $\varkappa_{1}=2.5\;\mathrm{GPa}/\mathrm{mm}$ one must see the gap by the modes $S_{0}$ and $S_{1}$ in frequency range $G_{1}$, and by the modes $S_{0}$ and $S_{5}$ in frequency range $G_{2}$ (see Figure 9b, Figure 10 and Figure 11). Here we consider only modes, which are observed in the experiment (see Section 5). Of course, analogous gaps could be observed for the other symmetric modes, if one could see the modes themselves.

### 3.5. Analysis of the Influence of the Film Parameters on the Basis of EBCs

If the goal is to consider plane LW propagating in $x_{1}$-direction, the EBCs (21) and (22) can be written in simpler form by setting $\partial /\partial x_{2}=0$$u_{2}=0$, $\sigma_{12}=\sigma_{32}=0$. Besides, in the case of a soft film one can use the simplified EBCs, obtained in the end of Section 3.5. Let us write them down in the form

(44) $$\begin{array}{c} {\hat{\sigma }}_{13}=-j_{1}h_{0}\frac{\nu_{2}}{1-\nu_{2}}\frac{\partial {\hat{\sigma }}_{33}}{\partial x_{1}}+j_{\mathrm{red}}h_{0}\rho_{2}\left[\frac{\partial^{2}{\hat{u}}_{1}}{\partial t^{2}}-j_{\mathrm{sim}}\frac{1}{2}\left(\xi_{1}^{\mathrm{eff}}-\xi_{1,0}^{\mathrm{eff}}\right)\frac{\partial^{2}{\hat{\sigma }}_{13}}{\partial t^{2}}\right],\;\\ {\hat{u}}_{3}=\frac{1}{2}\xi_{3}^{\mathrm{eff}}{\hat{\sigma }}_{33}-j_{1}h_{0}\frac{\nu_{2}}{1-\nu_{2}}\left[\frac{\partial {\hat{u}}_{3}}{\partial x_{1}}-j_{\mathrm{sim}}\frac{1}{2}\left(\xi_{1}^{\mathrm{eff}}-\xi_{1,0}^{\mathrm{eff}}\right)\frac{\partial {\hat{\sigma }}_{13}}{\partial x_{1}}\right]\\ -j_{\mathrm{sim}}\frac{h_{0}{\left(1-2\nu_{2}\right)}^{2}}{48{\left(1-\nu_{2}\right)}^{2}}{\left(\xi_{1,0}^{\mathrm{eff}}\right)}^{2}\rho_{2}\frac{\partial^{2}{\hat{\sigma }}_{33}}{\partial t^{2}} \end{array}$$

for symmetric LWs and

(45) $$\begin{array}{c} {\hat{\sigma }}_{33}=-j_{1}h_{0}\frac{\partial {\hat{\sigma }}_{13}}{\partial x_{1}}+j_{\mathrm{red}}h_{0}\rho_{2}\left[\frac{\partial^{2}\hat{u}}{\partial t^{2}}-j_{\mathrm{sim}}\frac{1}{2}\left(\xi_{3}^{\mathrm{eff}}-\frac{1-2\nu_{2}}{2(1-\nu_{2})}\xi_{1,0}^{\mathrm{eff}}\right)\frac{\partial^{2}{\hat{\sigma }}_{33}}{\partial t^{2}}\right],\;\\ {\hat{u}}_{1}=\frac{1}{2}\xi_{1}^{\mathrm{eff}}{\hat{\sigma }}_{13}-j_{1}h_{0}\left[\frac{\partial {\hat{u}}_{3}}{\partial x_{1}}-j_{\mathrm{sim}}\frac{1}{2}\left(\xi_{3}^{\mathrm{eff}}-\frac{1-2\nu_{2}}{2(1-\nu_{2})}\xi_{1,0}^{\mathrm{eff}}\right)\frac{\partial {\hat{\sigma }}_{33}}{\partial x_{1}}\right]\\ -j_{\mathrm{sim}}\frac{h_{0}}{12}{\left(\xi_{1,0}^{\mathrm{eff}}\right)}^{2}\rho_{2}\frac{\partial^{2}{\hat{\sigma }}_{13}}{\partial t^{2}} \end{array}$$

for antisymmetric ones, where $j_{\mathrm{red}}=0,1$ and $j_{\mathrm{sim}}=0,1$ are standing for the approximation type. In (44) and (45), the film-connection is characterized by five material parameters: $\xi_{1}^{\mathrm{eff}}$, $\xi_{3}^{\mathrm{eff}}$, $\nu_{2}$, $\rho_{2}$, $\xi_{1,0}^{\mathrm{eff}}$. By setting $j_{\mathrm{sim}}=0$, we come to reduced EBCs:

(46) $$\begin{array}{c} \mathrm{SLW}:\;\left\{\begin{array}{c} {\hat{\sigma }}_{13}=-j_{1}h_{0}\frac{\nu_{2}}{1-\nu_{2}}\frac{\partial {\hat{\sigma }}_{33}}{\partial x_{1}}+j_{\mathrm{red}}h_{0}\rho_{2}\frac{\partial^{2}{\hat{u}}_{1}}{\partial t^{2}},\\ {\hat{u}}_{3}=\frac{1}{2}\xi_{3}^{\mathrm{eff}}{\hat{\sigma }}_{33}-j_{1}h_{0}\frac{\nu_{2}}{1-\nu_{2}}\frac{\partial {\hat{u}}_{3}}{\partial x_{1}}, \end{array}\right.\\ \mathrm{ALW}:\;\left\{\begin{array}{c} {\hat{\sigma }}_{33}=-j_{1}h_{0}\frac{\partial {\hat{\sigma }}_{13}}{\partial x_{1}}+j_{\mathrm{red}}h_{0}\rho_{2}\frac{\partial^{2}{\hat{u}}_{3}}{\partial t^{2}},\\ {\hat{u}}_{1}=\frac{1}{2}\xi_{1}^{\mathrm{eff}}{\hat{\sigma }}_{13}-j_{1}h_{0}\frac{\partial {\hat{u}}_{1}}{\partial x_{1}}. \end{array}\right. \end{array}$$

The advantage of (46) consist in the fact, that they contain only four material parameters: $\xi_{1}^{\mathrm{eff}}$, $\xi_{3}^{\mathrm{eff}}$, $\nu_{2}$, $\rho_{2}$. Let us also write down the first order EBCs

(47) $$\begin{array}{c} \mathrm{SLW}:\;\;\left\{\begin{array}{c} {\hat{\sigma }}_{13}=-j_{1}h_{0}\frac{\nu_{2}}{1-\nu_{2}}\frac{\partial {\hat{\sigma }}_{33}}{\partial x_{1}},\\ {\hat{u}}_{3}=\frac{1}{2}\xi_{3}^{\mathrm{eff}}{\hat{\sigma }}_{33}-j_{1}h_{0}\frac{\nu_{2}}{1-\nu_{2}}\frac{\partial {\hat{u}}_{3}}{\partial x_{1}},\; \end{array}\right.\mathrm{ALW}:\;\;\left\{\begin{array}{c} {\hat{\sigma }}_{33}=-j_{1}h_{0}\frac{\partial {\hat{\sigma }}_{13}}{\partial x_{1}},\\ {\hat{u}}_{1}=\frac{1}{2}\xi_{1}^{\mathrm{eff}}{\hat{\sigma }}_{13}-j_{1}h_{0}\frac{\partial {\hat{u}}_{1}}{\partial x_{1}} \end{array}\right. \end{array}$$

with three parameters and the zero order EBCs

(48) $$\begin{array}{c} \mathrm{SLW}:\;{\hat{\sigma }}_{13}=0,\;\;{\hat{u}}_{3}=\frac{1}{2}\xi_{3}^{\mathrm{eff}}{\hat{\sigma }}_{33},\;\mathrm{ALW}:\;{\hat{\sigma }}_{33}=0,\;\;{\hat{u}}_{1}=\frac{1}{2}\xi_{1}^{\mathrm{eff}}{\hat{\sigma }}_{13} \end{array}$$

containing only two ones. As one can see from (44)–(47), the parameters $\nu_{2}$, $\rho_{2}$ arise only in the higher-order asymptotic approximations. It means that their estimation from the experimental data can be strongly affected by the noise and the other type of experimental errors. Thus, it is advisable to define the regions, where the influence of each of the parameters is the most pronounced. This can be done on the basis of the EBCs as follows.

Let us assume that we have two aluminuim plates of the thickness 2 mm, glued together by a film, and the experimentally acquired SCs of LWs. The material properties of the aluminuim can be determined by conducting an analogous experiment for a single plate, so we assume them to be known and coinciding with those given in Table 1. The thickness of the film is also assumed to be known and equal to 50 μm, but its material parameters must be extracted from the experimental data. Let us also suppose, that we are not sure whether the contact between the film and the aluminium is perfect or not. Therefore, we have to determine 5 parameters: $E_{2}$, $\nu_{2}$, $\rho_{2}$, $\xi_{1}$, $\xi_{3}$. This problem is equivalent to the evaluation of parameters $\xi_{1}^{\mathrm{eff}}$, $\xi_{3}^{\mathrm{eff}}$, $\nu_{2}$, $\rho_{2}$, $\xi_{1,0}^{\mathrm{eff}}$ entering in EBCs (44) and (45).

**Figure 12:**
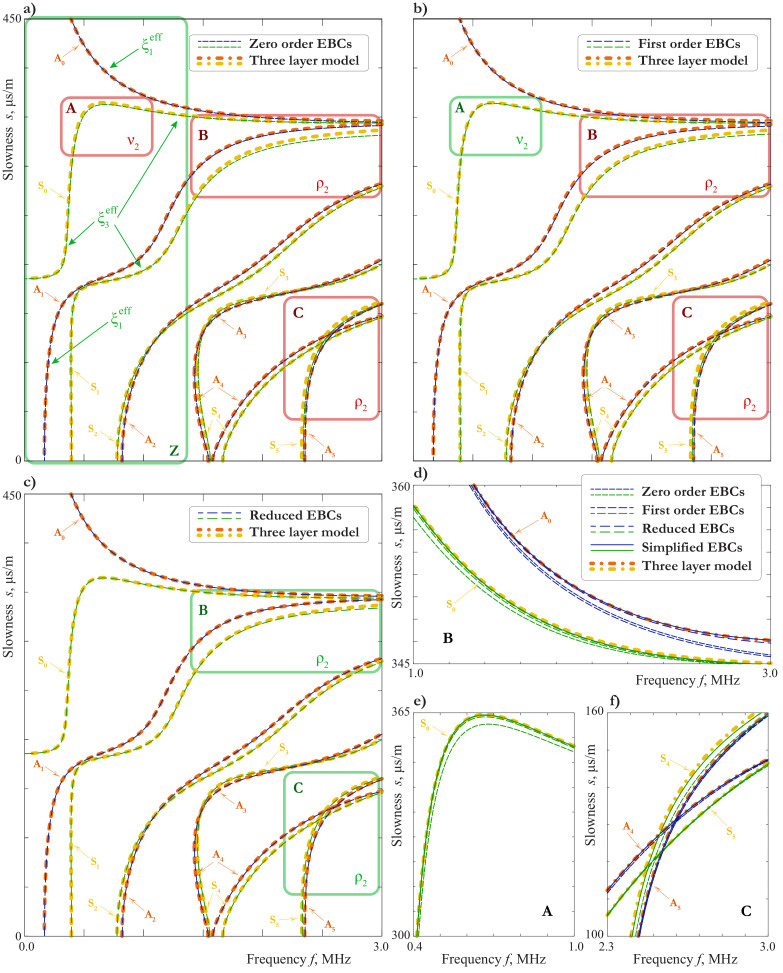
Slownesses of LWs propagating in 4.05 mm thickness plate (2 mm aluminium/50 μm film/2 mm aluminium) for different approximations of EBCs: (**a**)—zero-order EBCs (48), (**b**)—first order EBCs (47), (**c**)—reduced EBCs (46); (**d**–**f**)—zoomed regions B, A and C from subplots (**a**–**c**).

**Figure 13:**
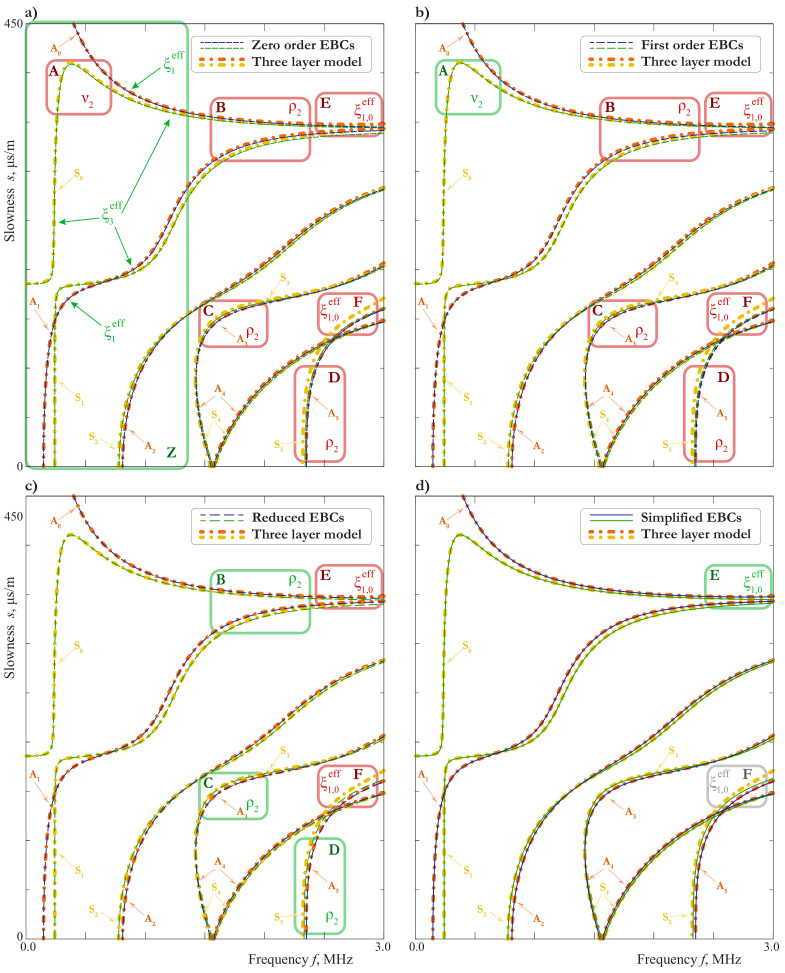
Slownesses of LWs propagating in 4.05 mm thickness plate (2 mm aluminium/50 μm film/2 mm aluminium) with an imperfect contact at the interfaces ($\varkappa_{1}=\varkappa_{3}=20\;\mathrm{GPa}/\mathrm{mm}$) for different approximations of EBCs: (**a**)—zero-order EBCs (48), (**b**)—first order EBCs (47), (**c**)—reduced EBCs (46), (**d**)—simplified EBCs (44) and (45).

At the first step, we calculate SCs with material parameters of a film, which is expected to be similar to the one used in the experiment (e.g., the two-side epoxy tape from the Table 1), using both the exact three layer model and the zero-order EBCs (48). By comparing the results, we can find region Z∖A in the slowness-frequency domain, where the SCs are well described, when using zero-order EBCs (see Figure 12a and Figure 13a). Analysis of the properties of Lamb waves presented in Section 3.1 allows to define modes within these regions, which are strongly influenced by the film. These modes are indicated by arrows in Figure 12a and Figure 13a. If the experimental data for these parts of SCs are available, one can determine parameter $\xi_{3}^{\mathrm{eff}}$ by matching the symmetric modes and $\xi_{1}^{\mathrm{eff}}$ for the antisymmetric ones. Notice, that these two parameters can be determined independently.

If the contact between layers is perfect, we can also find the Poisson’s ratio (PR) at this stage:

(49) $$\nu_{\mathrm{pr}}=\frac{\xi_{1}^{\mathrm{eff}}-2\xi_{3}^{\mathrm{eff}}}{2\left(\xi_{1}^{\mathrm{eff}}-\xi_{3}^{\mathrm{eff}}\right)}.$$

Let us call this a provisional Poisson’s ratio, since it is not valid in the case of an imperfect contact.

At the next step, we calculate the SCs using the first order EBCs with already known $\xi_{1}^{\mathrm{eff}}$ and $\xi_{3}^{\mathrm{eff}}$. The comparison shows that the influence of the new parameter $\nu_{2}$ consist mainly in improving the SC of the wave $S_{0}$ in the region A (see Figure 12a,b,e and Figure 13a,b). If we have the data for this region, we can choose an appropriate value of $\nu_{2}$, which is called an experimental PR. A discrepancy between the provisional PR and the experimental PR indicates that the contact is imperfect. For example, we have $\nu_{\mathrm{pr}}=0.19$ for $\varkappa_{1}=20$ GPa, $\varkappa_{3}=20$ GPa, $\nu_{\mathrm{pr}}=-0.05$ for $\varkappa_{1}=\infty$, $\varkappa_{3}=20$ GPa, and $\nu_{\mathrm{pr}}=0.43$ for $\varkappa_{1}=20$ GPa, $\varkappa_{3}=\infty$ instead of $\nu_{2}=0.4$ for the two-side epoxy tape. Thus, the provisional PR is not suitable in the case of an imperfect contact. However, the experimental PR is valid in both cases.

Now we are in the position to define the density by calculating the SCs using reduced EBCs (46), in which all the parameters except $\rho_{2}$ are known. The influence of $\rho_{2}$ is most pronounced in the regions B and C (see Figure 12b,c,d,f or B, C and D Figure 13b,c). At the last step, we determine the parameter $\xi_{1,0}^{\mathrm{eff}}$ by using the simplified EBCs (44) and (45) and matching the SCs in the regions E and F (see Figure 13c,d). If the accuracy of the simplified EBCs is not sufficient (as in the region F in Figure 13d), one can use tri-layer model and match the Young modulus $E_{2}$, since all the other parameters of that model can be expressed through it and already known quantities. On the each step beginning from the second one, an iteration procedure for the refinement of parameters is possible (e.g., after the determination of $\rho_{2}$ we can refine $\nu_{2}$ to meet the small changes between the first order and reduced EBCs in the region A (see Figure 12e), then, if necessary, refine $\rho_{2}$ to meet changes in B, C, and so on).

Thus, all the material parameters related to the film can be determined. In the case of a perfect contact $\xi_{1,0}^{\mathrm{eff}}=\xi_{1}^{\mathrm{eff}}$, i.e., all the material parameters are already known before the last step. In this case, the simplified EBC can be used to check the found parameters (see Figure 12d,e,f).

The consideration above shows that experimental data of high accuracy are needed to determine all the parameters of the film. If such data are not available, it is more reasonable to define the effective stiffnesses $\varkappa_{1}^{\mathrm{eff}}={\left(\xi_{1}^{\mathrm{eff}}\right)}^{-1}$, $\varkappa_{3}^{\mathrm{eff}}={\left(\xi_{3}^{\mathrm{eff}}\right)}^{-1}$ only. They allow to describe the SCs with a practically good accuracy and are sufficient to detect the damage of the interfaces between the film and the aluminium. In the case, when all the parameters are required, the step-wise algorithm presented above can be used to check, whether the amount and accuracy of the experimental data are sufficient to fulfil the task or not.

## 4. Properties of Other Guided Waves in Laminates with Soft Interlayer

Besides the Lamb waves, the laminate under consideration can guide horizontally polarized shear waves (SH-waves). The anti-plane problem describing them can be obtained for the general statement in Section 2.1 by setting $u_{1}=u_{3}=0$, $\frac{\partial }{\partial x_{2}}=0$. The corresponding EBCs follow from (14) or (21) and (22) after the same setting. Let us write down anti-plane EBCs for a symmetric laminate:

for the symmetric vibrations:

(50) $${\hat{\sigma }}_{23}=-j_{2}h_{0}\mu_{2}\Omega_{\mathrm{sh}}\left({\hat{u}}_{2}-\xi_{2}{\hat{\sigma }}_{23}\right),$$

for the antisymmetric vibrations:

(51) $${\hat{u}}_{2}=\frac{1}{2}\xi_{2}^{\mathrm{eff}}{\hat{\sigma }}_{23}+j_{2}\frac{h_{0}^{3}}{3\mu_{2}}\Omega_{\mathrm{sh}}{\hat{\sigma }}_{23},$$

where $\Omega_{\mathrm{sh}}=\frac{\partial^{2}}{\partial x_{1}^{2}}-\frac{1}{c_{2,T}^{2}}\frac{\partial^{2}}{\partial t^{2}}$, $\xi_{2}^{\mathrm{eff}}$ is defined by (17). As one can see from (50), the symmetric SH-wave coincides with some SH-wave of the single layer with asymptotic error of the second order. With the same error, the antisymmetric SH-wave is SH-mode of the same layer with elastic constraint on the bottom surface, defined by the effective stiffness $\varkappa_{2}^{\mathrm{eff}}={\left(\xi_{2}^{\mathrm{eff}}\right)}^{-1}$ (see (51)). If this stiffness is sufficiently small, we have a long-wave, low-frequency mode with non-zero cut-off frequency. This case is thoroughly studied in [41].

The properties of SH-waves in the laminate are analogous to those of LWs analyzed in Section 3.1, but in this case the symmetric modes are not affected by the stretch interlayer parameter. In the long-wave range in respect to film ($h_{2}\ll L$), all the SH-modes can be considered as a symmetric or antisymmetric couple of symmetric or antisymmetric SH-waves of the upper (or the lower) layer, with an exception of the mode $ASH_{0}$. The latter represents an antisymmetric couple of symmetric modes ${\mathrm{ssh}}_{0}$ at high frequencies, but at low frequencies it behaves differently and has non-zero cut-off frequency.

The form of EBCs (50) and (51) shows, that in their range of applicability the dispersion curves of antisymmetric SH-waves depend mainly on the parameter $\xi_{2}^{\mathrm{eff}}$. The dependence on the shear modulus $\mu_{2}$ in particular is defined by the second-order asymptotic term, so it must be very weak. If the stiffness $\varkappa_{2}=\xi_{2}^{-1}$ is not too small, the dispersion curves of symmetric SH-waves depend weakly on $\mu_{2}$, $\rho_{2}$ and $h_{2}$. In the case $\mu_{2}\ll \mu_{1}$, the first term in $\Omega_{\mathrm{sh}}$ is small comparing to the second one, so the dispersion curves are not sensitive to $\mu_{2}$. These conclusions are in agreement with the results of paper [10], where a similar problem was considered and the non-sensitivity of the mode ${\mathrm{SH}}_{2}$ (${\mathrm{SSH}}_{1}$ in the notations of the present paper) to $\mu_{2}$ and $\varkappa_{2}$ was revealed by means of numerical FE-based investigation. Thus, in the range of applicability of EBCs (50) and (51) it is hardly possible to extract both $\mu_{2}$ and $\varkappa_{2}$ from the experimentally acquired dispersion properties of SH-waves.

As for LWs, the EBCs (50) and (51) fail in the vicinities of the thickness resonance frequencies of the film. On the basis of the three layer model, the latter are defined by Equations (27), (29) and (31) with $\varkappa_{2}$ instead of $\varkappa_{1}$. The lowest of such frequencies is observed for symmetric modes. Only if this frequency comes to be in the frequency range under consideration, one can find both $\mu_{2}$ and $\varkappa_{2}$. Thus, even in the best case, SH-waves allow to determine only three parameters of the film: the shear modulus, the density and the interface stiffness in tangential direction. So it is of interest to study the possibilities of the other GWs.

Let us consider a semi-infinite laminate, occupying the domain $-\infty <x_{1}<\infty ,$ $x_{2}⩽0,0⩽x_{3}⩽h$ (see Figure 1). In this case, the plate can support one more type of GWs—edge waves (EWs), propagating along the edge $x_{2}=0$ in $x_{1}$ direction and exponentially decaying as $x_{2}\to -\infty$. These waves were intensively studied theoretically (see the overview [42] and the references therein), in the recent time their existence and properties were confirmed in several experimental studies [43,44,45,46]). But the EWs in a laminate glued by a thin soft film were not yet investigated.

In this work, we consider a symmetric laminate with perfect contact on the interfaces, and employ the second order EBCs (21) and (22). The problem is reduced to one for the upper layer with free top surface, EBCs (21) or (22) at the bottom surface, and BCs on the edge $x_{2}=0$

(52) $$\sigma_{l2}^{\left(1\right)}=q_{l}\left(x_{1},z,t\right),\;\int_{0}^{h}\sigma_{l2}^{\left(1\right)}dz+\int_{0}^{h_{0}}\sigma_{l2}^{\left(2\right)}dy=\int_{0}^{h}q_{l}\left(x_{1},z,t\right)dz,$$

where l=1,2,3, $q_{l}\left(x_{1},z,t\right)$ are prescribed loads. In (52), $\sigma_{l2}^{\left(2\right)}$ are stresses in the film, which can be calculated with the asymptotic error $O\left(\varepsilon^{3}\right)$ as stated in Section 2.2 after deriving the EBCs. We assume that the film is unloaded, and require the satisfying of edge BC for the film in the integral form only, which is justified for the case of the long-wave vibrations ($L\gg h_{2}$).

This statement of the problem is analogous to that one considered in [43], so one can apply the same method, which is based on the use of the Laplace and the Fourier integral transforms and expansion through wave modes of the infinite layer. As in [43], both LWs and SH-waves must be taken into account. The unknown constants of the expansion are determined by satisfying BC (52) as described in [43].

**Figure 14:**
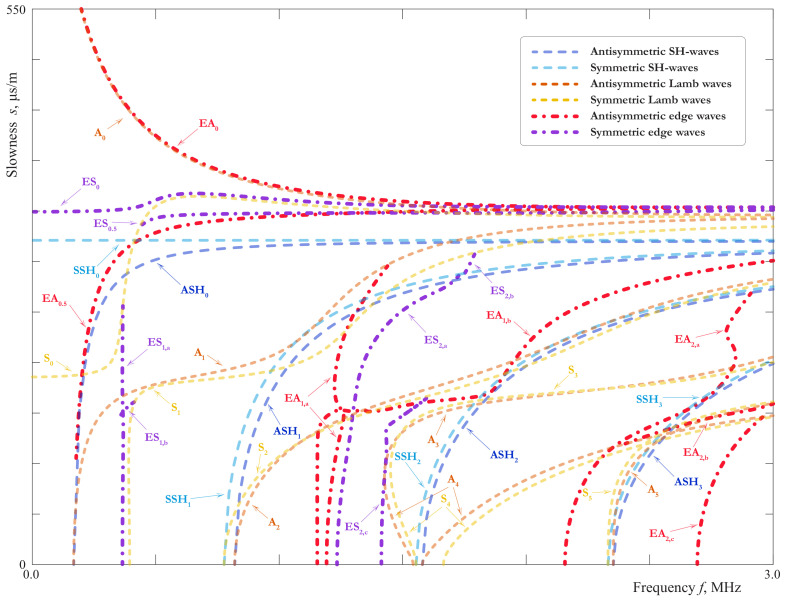
Slownesses of all GWs propagating in 4.05 mm thickness symmetric laminate with a soft thin interlayer (2 mm aluminium/50 μm film/2 mm aluminium).

**Figure 15:**
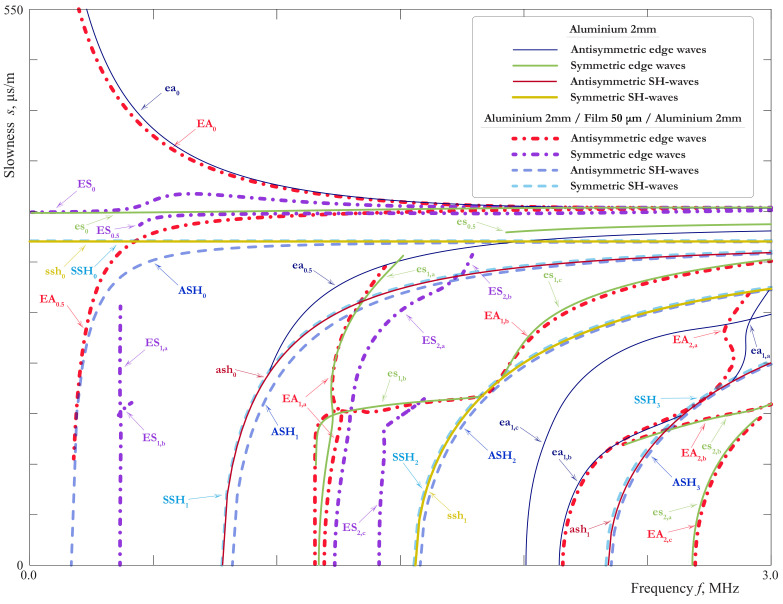
Slownesses of EWs and SH-waves propagating in 4.05 mm thickness symmetric laminate with a soft thin interlayer (2 mm aluminium/50 μm film/2 mm aluminium) and 2 mm aluminium plate.

EWs correspond to poles $\omega_{m}\left(k\right)$ (*k* is the wavenumber) in the complex plane ω, which are found numerically. The calculated slownesses of EWs are shown in Figure 14 and Figure 15 together with the slownesses of LWs and SH-waves. The notations ${\mathrm{EA}}_{n}$, ${\mathrm{ES}}_{n}$, introduced for a homogeneous plate, are applicable to a symmetric laminate as well. The SCs for an aluminium plate of the thickness 2 mm are also shown here for comparison.

**Figure 16:**
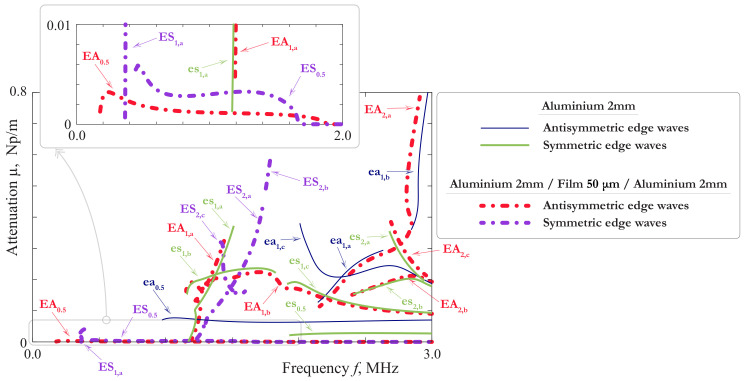
Attenuation of EWs propagating in 4.05 mm thickness symmetric laminate with a soft thin interlayer (2 mm aluminium/50 μm film/2 mm aluminium) and 2 mm aluminium plate.

The attenuation of EWs defined as Imωm(k) is shown in Figure 16. This effect is caused by the radiation of the energy transferred into the interior of the plate due to the coupling of EWs with propagating LWs and SH-waves. It is characteristic for edge modes with attenuation that their dispersion curves split into branches because of the intersection with the cuts in the complex plane, associated with propagating Lamb and SH-modes.

The behaviour of the SCs for SH-waves is demonstrated in Figure 14 and their comparison with the SH-waves in the 2mm-thick aluminium homogeneous layer is shown in Figure 15. It confirms the results of the theoretical analysis given above. Except ${\mathrm{ASH}}_{0}$, all the SH-waves of the laminate are only slightly influenced by the film. The behaviour of EWs is more complicated. As one can see from the Figure 14 and Figure 15, in the laminate with a soft thin interlayer one can observe a richer family of EWs than in a monolithic layer. In general, it reproduces the main properties of LWs investigated n Section 3.1. There are the pairs of SCs in Figure 14 and Figure 15 corresponding to SCs of EWs in a 2mm-thick aluminium layer, although the EWs associated with ${\mathrm{ea}}_{0.5}$, ${\mathrm{es}}_{0.5}$ and symmetric waves associated with ${\mathrm{ea}}_{1}$ were not found. Apparently, the influence of the film has moved the corresponding poles to the hidden sheets of the Riemann surface. It is interesting to notice that the high order EWs ${\mathrm{EA}}_{0.5}$, ${\mathrm{ES}}_{0.5}$, ${\mathrm{ES}}_{1}$ in their main features are close to fundamental waves: their SCs are in general lay close together, and their cut-off frequencies and attenuation is small. To our best knowledge, such type of EWs, which could be called quasi-fundamental EWs, was not studied before. The other higher order EWs revealed in this paper are better observed in a thick plate, as it was shown in [44].

The most interesting from the practical point of view are fundamental waves ${\mathrm{EA}}_{0}$, ${\mathrm{ES}}_{0}$ and theirs pairs ${\mathrm{EA}}_{0.5}$, ${\mathrm{ES}}_{0.5}$, ${\mathrm{ES}}_{1}$, which are most likely to be observed in the experiments. The analogy with LWs allows to suggest that they can provide the information about $\xi_{1}^{\mathrm{eff}}$, $\xi_{3}^{\mathrm{eff}}$ and, in the case of highly accurate experimental data, about $\nu_{2}$ and $\rho_{2}$. However, the possibility of the evaluation of $\mu_{2}$ and $\xi_{1}$ taken separately is rather questionable, unless the available frequency range contains regions, where the long-wave EBCs are not valid because of some resonance phenomena in the film. In the latter case, EWs have an advantage in comparison with LWs and SH-waves. As it is shown in [43], EWs are well observed by measurements on the edge, where one can acquire the wave-field in the neighbourhood of the film, and so obtain more information about dynamic behaviour of the latter, than from data acquired on the faces of the laminate. The investigation of EWs on the basis of the three layer model would make this paper too voluminous, so it will be the topic of the future work.

## 5. Comparison: Theory vs. Experiment

### 5.1. Experimental Setup

**Figure 17:**
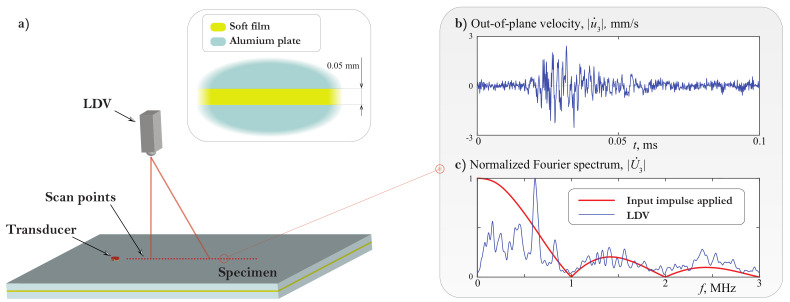
Sketch of the experimental setup (**a**). Out-of-plane velocities (**b**) and their spectrum (**c**) measured by the LDV at a point located 70 mm away from the piezoelectric actuator center after its broadband excitation with 1 μs rectangular pulse tone burst voltage.

To verify the predicted properties experimentally, a three-layered specimen was fabricated of two 2 mm-thickness aluminium plates of dimensions 600×150×2 mm${}^{3}$ joined by an double sided adhesive film (acquired from selbstklebefolien.com) of 50 μm thickness as shown in Figure 1 and Figure 17a. The resulting laminates were further cured for 24 h at room temperature under uniform pressure of 2000 Pa.

GWs in the specimen are excited by a thin adhesively attached circular piezoelectric actuator of 5 mm radius and 0.5 mm thickness manufactured from PZT PIC 151 (PI Ceramic GmbH, Germany). Out-of-plane velocities of propagating wave packages are acquired in a non-contact manner on the specimen surface by PSV-500-V laser Doppler vibrometer (LDV) (Polytec GmbH, Waldbronn, Germany), which head is placed about 1100 mm above the sample minimizing the oblique angle laser beam measurement errors [47]. Since the specimen surfaces remained intact with no special treatment for their reflectivity improvement applied, at least 200 time averagings are performed for each measurement point to improve signal-to-noise ratio. Moreover, 3 MHz low-pass filtering is introduced with LDV software and 7.8125 MHz sampling frequency is chosen to meet the Nyquist criterion. The scheme of the experimental setup is shown in Figure 17a.

To illustrate the capability of broadband LW excitation with the employed piezoelectric actuator which is essential for further evaluation of experimental dispersion curves, a typical LDV-acquiered wave signal and its spectrum are shown in Figure 17b,c. The actuator was driven by broadband 1 μs rectangular pulse tone burst voltage which spectrum is also provided in Figure 17c (red curve). The excited wave signal covers the proposed frequency range up to 3 MHz, and, as expected, is close to zero only at local minima of the driving tone burst at 1, 2 and 3 MHz.

### 5.2. Analysis of the Experimental Data

**Figure 18:**
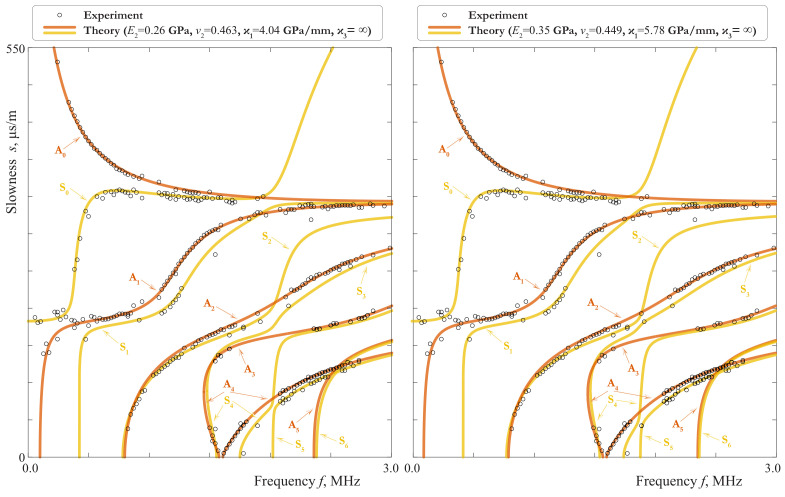
Slownesses of LWs propagating in 4.05 mm thickness plate (2 mm aluminium/50 μm film/2 mm aluminium) determined via the MPM (circles) and estimated theoretically (solid lines).

Experimental slownesses for the fabricated laminate structure occupying in the introduced cooridinate system the domain $|x_{1}|<300,-150<x_{2}<0,0<x_{3}<4.05$ are shown in Figure 18 by circles. These slownesses have been computed applying the matrix pencil method (MPM) [48] to out-of-plane velocities measured along the interval $20\leq x_{1}\leq 180$ mm, $x_{2}=-75$ mm, $x_{3}=4.05$ mm with 0.3 mm step after piezoelectric actuator excitation with 1 μs rectangular pulse tone burst voltage. As it can be seen from Figure 18, the MPM-data are in a good agreement with theoretically calculated slownesses for the three-layered laminate. In particular, one can see the pairs of dispersion curves laying closely together ($A_{0}$ and $S_{0}$, $A_{1}$ and a part of $S_{1}$, $A_{4}$ and $S_{4}$), which were predicted and explained in the theoretical part of this investigation.

The material parameters, used for theoretical SCs in Figure 18, were determined as follows. Preliminary, we refined the parameters of the aluminum layers in an analogous experiment for a single 2mm-thick plate before gluing. The material properties of aluminium plate are shown in the Table 2. Notice, that the experimental data for the laminate itself can be also used to refine the parameters of the aluminium. It was shown in Section 3.1 that the SCs of modes $A_{1}$ in the range 1.1–1.3 MHz, $A_{2}$ and $A_{4}$ in the range 1.5–2.7 MHz are nearly coincident with those of a single 2mm-thick aluminium layer, and these stretches are well observed in MPM-data. However, these parts of experimental data are useless, when the goal is to determine the parameters of the film. Let us consider the data, which cannot be described by SCs of an aluminium plate, and proceed according to the procedure given in Section 3.5.

At the first step, we find the values of the effective stiffnesses ${\left(\xi_{1}^{\mathrm{eff}}\right)}^{-1}=\varkappa_{1}^{\mathrm{eff}}=$ 1–1.2 GPa/mm, ${\left(\xi_{3}^{\mathrm{eff}}\right)}^{-1}=\varkappa_{3}^{\mathrm{eff}}=$ 24.5–27.5 GPa/mm by fitting the experimental data in the region Z\A with the SCs, calculated on the basis of the model with zero-order EBC. The provisional Poisson’s ratio (49) changes in the limits $\nu_{\mathrm{pr}}=$0.474–0.481 and seems to be too high for this material. Since $\nu_{\mathrm{pr}}$ increases when $\xi_{1}^{\mathrm{eff}}$ grows, we can suggest that the film-aluminium interface has some compliance in the tangential direction. At the second step, we can only say that $\nu_{2}=$ 0.3–0.5, since the divergence of the MPM-data is too large to find the Poisson’s ratio more definitely. But the influence of $\nu_{2}$ is negligible in the region B, so we can define the density as $\rho_{2}=$ 800–1000 $\mathrm{kg}/m^{3}$. For the last step, there are no data in the regions D and E, so it is impossible to determine the last parameter $\xi_{1,0}^{\mathrm{eff}}$. It means, that the experimental data are insufficient to define the Young modulus of the film and the stiffnesses of the interfaces. However, with the use of some additional considerations we can deduce the limits of the estimated values of $E_{2}$ and $\nu_{2}$.

Taking into account the symmetry, we express from (17)

(53) $$\xi_{1}=\frac{1}{2}\xi_{1}^{\mathrm{eff}}-\frac{h_{2}\left(1+\nu_{2}\right)}{E_{2}},\;\xi_{3}=\frac{1}{2}\xi_{3}^{\mathrm{eff}}-\frac{h_{2}\left(1+\nu_{2}\right)\beta_{2}^{2}}{E_{2}}.$$

From the condition $\xi_{1}⩾0$, $\xi_{3}⩾0$ follows that the pair $\left(E_{2},\nu_{2}\right)$ must lay in the domain $P_{1}\cap P_{3}$ with $P_{1}:\;E_{2}⩾2h_{2}\left(1+\nu_{2}\right)\varkappa_{1}^{\mathrm{eff}}$, $P_{3}:\;E_{2}⩾2h_{2}\left(1+\nu_{2}\right)\beta_{2}^{2}\varkappa_{3}^{\mathrm{eff}}$. Besides, from (53) follows $\varkappa_{1}=\xi_{1}^{-1}>2\varkappa_{1}^{\mathrm{eff}}$, $\varkappa_{3}=\xi_{3}^{-1}>2\varkappa_{3}^{\mathrm{eff}}$. Applying these inequalities together with formulas (26) and (32), we obtain estimations

(54) $$f_{\mathrm{fl},\mathrm{st}}^{a}>f_{\mathrm{fl},0}^{a}\;f_{\mathrm{fl},\mathrm{sh}}^{s}>f_{\mathrm{fl},0}^{s},$$

where $f_{\mathrm{fl},0}^{a}$ is the root of Equation (23) at $\varkappa_{3}=2\varkappa_{3}^{\mathrm{eff}}$, $\mu_{2}/\beta_{2}^{2}\to \infty$, which is the nearest to the approximate value (26) as $\varkappa_{3}\to 0$. Analogously, $f_{\mathrm{fl},0}^{s}$ is the root of Equation (29) at $\varkappa_{1}=2\varkappa_{1}^{\mathrm{eff}}$, $\mu_{2}\to \infty$, which is the nearest to the approximate value (32) as $\varkappa_{1}\to 0$. For the following considerations, let us assume the mean values for $\varkappa_{1}^{\mathrm{eff}}$, $\varkappa_{3}^{\mathrm{eff}}$, $\rho_{2}$, given in Table 2, as the experimentally determined parameters of the two-side epoxy type. Then we have $f_{\mathrm{fl},\mathrm{st}}^{a}>7.5$ MHz, which is far outside the frequency limit of the experimental data. But for the symmetric shear resonance frequency, the estimation (54) gives $f_{t}^{\mathrm{fl}}>1.6\;\mathrm{MHz}$. Theoretically, we could observe the effect of this resonance as gaps in SCs of symmetric modes, if $f_{\mathrm{fl},\mathrm{sh}}^{s}<3\;\mathrm{MHz}$. In our experimental data, we see the gaps by all modes around 1 MHz, 2 MHz and 3 MHz. Apparently, they are related to the spectrum of the pulse load with the duration 1μs. But the situation, when the frequency $f_{t}^{\mathrm{fl}}$ comes to be in one of the load-gaps around 2 MHz and 3 MHz, cannot be excluded. Starting from the fact, that we can see mode $S_{0}$ up to 1.75 MHz and mode $S_{4}$ in the range 2.08–2.7 MHz, and taking into account the width of the gaps shown in Figure 9, we obtain estimations for the possible values of $f_{\mathrm{fl},\mathrm{sh}}^{s}$:

(55) $$f_{\min,1}<f_{\mathrm{fl},\mathrm{sh}}^{s}<f_{\max,1},\;f_{\mathrm{fl},\mathrm{sh}}^{s}>f_{\min,2}$$

with $f_{\min,1}=1.88$ MHz, $f_{\max,1}=2.03$ MHz, $f_{\min,2}=2.8$ MHz. The numerical solving of Equation (29) with $\varkappa_{1}=\xi_{1}^{-1}$ defined by (53), allows to determine the domains $Q_{1}$, $Q_{2}$ of the possible values of $\left(E_{2},\nu_{2}\right)$, for which the inequalities (55) are satisfied. Thus, the pair $\left(E_{2},\nu_{2}\right)$ can lay in the domains $P_{1}\cap P_{3}\cap Q_{1}$ or $P_{1}\cap P_{3}\cap Q_{2}$. For the $\varkappa_{1}^{\mathrm{eff}}$ given in the Table 2, we found $P_{1}\cap P_{3}\cap Q_{2}=⌀$. By approximating the boundaries of $P_{1}\cap P_{3}\cap Q_{1}$, we come to the limits for possible values of $E_{2}$ and $\nu_{2}$, given in Table 2 (in the formula for the lower limit of $\nu_{2}$, *E* means the value of the Young modulus in GPa). According to formulas (53), the values of $\varkappa_{1}^{\mathrm{eff}}$, $\varkappa_{3}^{\mathrm{eff}}$, $E_{2}$, $\nu_{2}$ define the interface stiffnesses:

$$\varkappa_{1}^{\exp}=\frac{1}{\frac{1}{2}{\left(\varkappa_{1}^{\mathrm{eff}}\right)}^{-1}-\frac{h_{2}\left(1+\nu_{2}^{\exp}\right)}{E_{2}^{\exp}}},\;\;\varkappa_{3}^{\exp}=\frac{1}{\frac{1}{2}{\left(\varkappa_{3}^{\mathrm{eff}}\right)}^{-1}-\frac{h_{2}\left(1+\nu_{2}^{\exp}\right)\left(1-2\nu_{2}^{\exp}\right)}{2E_{2}^{\exp}\left(1-\nu_{2}^{\exp}\right)}},$$

where $E_{2}^{\exp}$, $\nu_{2}^{\exp}$ are some values from the ranges given in Table 2, $\varkappa_{1}^{\mathrm{eff}}$, $\varkappa_{3}^{\mathrm{eff}}$ are experimental values, also given in this table. The range of possible values of $E_{2}^{\exp}$ and $\nu_{2}^{\exp}$ corresponds to ranges $\varkappa_{1}^{\exp}\in \left[4,6\right]$ GPa/mm and $\varkappa_{3}^{\exp}\in \left[52,\infty \right)$ GPa/mm.

## 6. Discussion

With extensive analytical and numerical analysis, it is illustrated that mechanical properties of the thin soft interlayer and the interface contact quality have a sufficient influence on the EGWs properties in a three-layered laminate structures. Physically, it manifests itself in the occurrence of normal modes being related to the global structure or to the corresponding LWs of sublayers, emergence of repulsion effects and additional thickness resonances. Such impact can be efficiently described both quantitatively and qualitatively by the derived EBCs, where analytical expressions are now available for the expansion terms. Employing EBCs it becomes possible to provide physically clear explanation to the observed behaviour of high-order EGWs in considered laminate structures (i.e., emergence of mode pairs, closed-form representations for cut-off frequencies, etc.). Moreover, specific frequency regions and EGWs being most sensitive to interlayer mechanical properties and its bonding quality with external lamina are revealed. Therefore, a consequential procedure for soft interlayer identification based on the EBCs can be implemented using experimentally evaluated EGW dispersion curves obtained from the measurements on the specimen surface.

It is revealed that for a broad frequency range the interlayer influence on the elastodynamic behaviour of the laminate structure could be reliably described by just the effective stiffnesses $\varkappa_{1}^{\mathrm{eff}}$ and $\varkappa_{3}^{\mathrm{eff}}$ being a combination of the elastic moduli of the film, its thickness and interface stiffnesses. They could be reliably identified from experimental data and might be already used in certain NDT/SHM applications for contact integrity evaluation. For example, if the values of $\varkappa_{1}^{\mathrm{eff}}$ and $\varkappa_{3}^{\mathrm{eff}}$ are estimated in advance for a reference pristine structure, their deviation from baseline values indicates the changes either in contact condition or interlayer degradation. However, for some other practical applications the mechanical properties of the film itself may be essential, as well as the interface stiffnesses. In this case, one must take into account that the separate determination of these parameters involves higher order terms of EBCs, which have small influence on EGW behaviour. Therefore, special attention should be paid, whether the amount and accuracy of the available experimental data is sufficient to determine all the required parameters. For instance, with the experimental dispersion curves for LWs mentioned above, it is possible to provide unique output only if the thickness of the film is known in advance and the perfect contact is assured. If (as in the example considered) the last condition cannot be met for sure, the unique quantification of the elastic moduli and the interface stiffnesses turned out to be practically impossible. This result has its physical explanation in a well known fact that dynamic effects in a *thin* film have correspondingly *high* frequencies, which are hard to achieve in the current experiment. At lower frequencies the behaviour of the film is rather quasi-static, thus, not all of its parameters are equally involved in the dynamics of the laminate. Still, the theoretical analysis shows that from the complete and precise experimental data all the film-related parameters could be determined.

The peculiar property of the thin and *soft* film is that the lowest of its thickness resonance frequencies can be found in the low-frequency range, available for the experimental investigation. In this case, some additional information about the film-related parameters can be obtained, even if these frequencies could be observed only as gaps in the experimentally acquired dispersion curves. In the present paper, ranges of mutual variation of film and contact parameters are estimated via the consideration of possible values of film-related thickness resonance frequencies. As a general recommendation, it can be noticed that the broadening of the considered frequency range to include the resonance phenomena in the film is the best way to achieve unique determination of its parameters. In this regard, the EWs seem to be perspective candidate, since they allow observation on the edge in the vicinity of the film.

Although numerical examples and experimental validation are considered in this paper for a symmetric waveguide only, the employed computational model and derived effective boundary conditions (14) are valid for a laminate structure with dissimilar isotropic external layers of arbitrary thickness. Therefore, a general case of a non-symmetric three-layered laminate with soft thin film can be also efficiently investigated employing the analytical relations of the derived EBCs.

An example provided in this contribution demonstrates that though some characteristics of the laminate could be determined, a certain number of limitations exist. The limits of applicability of the results follow from the assumptions, made during the investigation. The EBCs are applicable in the case when the characteristic wavelength is much greater that the thickness of the film, and are suitable for frequency ranges outside the small vicinity of the thickness resonances of the film. The step-wise procedure for the evaluation of the film parameters assumes that the material of the film is homogeneous, isotropic and non-viscous. Moreover, it is assumed that the contact quality is uniform within the whole interface so that it could be described with a finite set of constants used in SBCs. Another limitation is that LDV scans over a single line are used in the presented study for material properties identification. High-amplitude reflections induced due to the presence of inhomogeneities near the scan line such as specimen edges, internal macroscopic localised defects, etc., may sufficiently spoil the data, and, therefore, sufficiently complicate the procedure of mechanical property identification if not being avoided.

For further research endeavors, it is essential to address viscoelastic behaviour of the interlayer typical for polymer-based materials in a three-layered model (Section 2.1) and EBCs and to investigate its influence on fundamental and high-order EGWs [49]. Another topic of emerging interest is the extension of the proposed methodology to metal-composite and composite-composite bonded structures [50] considering anisotropic mechanical properties of sublayers. The current study mainly concentrated on the investigation of LWs propagation. Although SH-waves and EWs, generally speaking, behave similarly, they might provide additional data for identification procedures (see an example of SH-waves employment in [10]) including those based on the derived EBCs. Therefore, further experimental and theoretical investigations related to the laminates with thin interlayers should also exploit the potential of EGWs of other kinds.

## Figures and Tables

**Table 1:** Material properties used for numerics.

Material	Density	Young Modulus	Poisson’s Ratio
	$\rho ,\mathrm{kg}/m^{3}$	*E*, GPa	ν
Aluminium	2700	70	0.33
Cyanoacrylate adhesive [34]	1248	1.7	0.4
Silicone rubber [35]	1150	3.1	0.48
Two-component epoxy adhesive [36]	1345	2.75	0.35
Two-sided epoxy tape [37]	930	0.5	0.4

**Table 2:** Material properties found experimentally.

Material	Eff. Stiffness		Density	Young Modulus	Poisson’s Ratio
	GPa/mm		ρ, $\mathrm{kg}/m^{3}$	*E*, GPa	ν
	$\varkappa_{1}^{\mathrm{eff}}$	$\varkappa_{3}^{\mathrm{eff}}$			
Aluminium	–	–	2715	72	0.345
Two-sided epoxy tape	1.1	26	900	0.26–0.35	$\left[0.505-\frac{0.16\;E}{\mathrm{GPa}}\right]$–0.5

## Data Availability

Data sharing not applicable to this article as no datasets were generated or analyzed during the current study.

## Abbreviations

The following abbreviations are used in this manuscript:

NDT	non-destructive testing
SHM	stuctural health monitoring
SCs	slowness curves
EGWs	elastic guided waves
GWs	guided waves
LWs	Lamb waves
ALW	antisymmetric Lamb wave
SLW	symmetric Lamb wave
EWs	edge waves
BCs	boundary conditions
EBCs	effective boundary conditions
SBCs	spring-type boundary conditions
LDV	laser Doppler vibrometer
